# A review of the spider genus *Haplodrassus* Chamberlin, 1922 in Crimea (Ukraine) and adjacent areas (Araneae, Gnaphosidae)

**DOI:** 10.3897/zookeys.205.3491

**Published:** 2012-07-04

**Authors:** Mykola M. Kovblyuk, Zoya A. Kastrygina, Mikhail M. Omelko

**Affiliations:** 1 Zoology Department, V.I. Vernadsky Taurida National University, Yaltinskaya str. 4, Simferopol 95007, Crimea, Ukraine; 2 Zoological Museum, University of Turku, FI-20014 Turku, Finland; 3 Gornotaezhnaya Station FEB RAS, Gornotaezhnoe Vil., Ussuriyski Dist., Primorski krai 692533 Russia; 4 Far Eastern Federal University, Sukhanova 8, Vladivostok 690950 Russia

**Keywords:** *Haplodrassus*, Crimea, Ukraine, Azerbaijan, Kazakhstan, redescriptions, fauna, phenology

## Abstract

Eight species of *Haplodrassus* are recorded from Crimea: *Haplodrassus bohemicus* Miller & Buchar, 1977; *Haplodrassus dalmatensis* (L. Koch, 1866); *Haplodrassus isaevi* Ponomarev & Tsvetkov, 2006; *Haplodrassus minor* (O. P.-Cambridge, 1879); *Haplodrassus kulczynskii* Lohmander, 1942; *Haplodrassus pseudosignifer* Marusik, Hippa & Koponen, 1996; *Haplodrassus signifer* (C.L. Koch, 1839) and *Haplodrassus umbratilis* (L. Koch, 1866). The occurrence of *Haplodrassus cognatus* (Westring, 1861) in Crimea has not been confirmed. *Haplodrassus bohemicus* is a new species record for the Crimean fauna. *Haplodrassus pseudosignifer* is a new species record for Crimea and Ukraine as a whole, with Crimea as the westernmost point of its distribution range. *Haplodrassus invalidus* is recorded for the first time for the fauna of Azerbaijan, Caucasus and the former Soviet Union. Azerbaijan is the easternmost point of its known distribution range. All Crimean *Haplodrassus* species have only one peak of activity of adult specimens during the year. In Crimea we found syntopically two closely related species *Haplodrassus dalmatensis* and *Haplodrassus isaevi* in two localities (Sudak Distr., 10 km W Sudak, Mezhdurechie Vill., steppe; and Feodosiya Distr., Karadag Nature Reserve, steppes). These species differ in their phenology. The reproductive period of *Haplodrassus dalmatensis* isin May-July, and that of *Haplodrassus isaevi* occurs is in October-December. These phenological differences probably represent an additional mechanism of reproductive isolation between the two species. Diagnostic drawings are provided for all mentioned species as well as for *Haplodrassus deserticola* Schmidt & Krause, 1996 and *Haplodrassus pugnans* (Simon, 1880).

## Introduction

*Haplodrassus* Chamberlin, 1922 with 65 species is a relatively large gnaphosid genus distributed in the Holarctic and India (Platnick 2012). The genus has been well revised for species that occur in North America (9 species) (Platnick and Shadab 1975), Central Europe (10 species) (Grimm 1985), Israel (8 species) (Levy 2004, 2009), China (8 species) (Song et al. 2004) and Japan (6 species) (Kamura 2007). However, in the Mediterranean region and Central Asia, the genus remains poorly studied.

In the former Soviet Union 21 species have been recorded to date (Mikhailov 1997; Tuneva 2005; Ponomarev and Tsvetkov 2006; Marusik et al. 2007; Ponomarev 2008; Ponomarev et al. 2008), with six of them reported from Crimea (Kovblyuk 2006, 2011). While identifying gnaphosid material collected in Crimea during the last few years we recognized two additional species. During our research to identify them we studied *Haplodrassus* species known from adjacent territories and found one species new to the fauna of Caucasus. Some of the species occurring in Crimea are relatively poorly known, and/or closely resemble widespread species. Therefore, this paper aims to illustrate all of the species found in the Crimea and those from adjacent territories. In addition, we provide data for the distribution and seasonal activity dynamics of adult *Haplodrassus* specimens in Crimea, a key to all Crimean species.

## Material and methods

Microphotographs were made using an SEM Jeol JSM-5200 in the Zoological Museum, University of Turku, Finland. Photographs were taken in dishes of different sizes with paraffin at the bottom. Specimens were photographed using an Olympus Camedia E-520 camera attached to an Olympus SZX16 stereomicroscope at the Zoological Museum, University of Turku. Digital images were montaged using “CombineZM” image stacking software.

Coloration was described from specimens preserved in an ethanol/water solution. Leg segments were measured after detaching them from the cephalothorax. All measurements are in mm: minimum-maximum; a figure in brackets represents the average. Illustrations were made using both reflecting- and transmitted-light microscopes. All scale bars equal 0.1 mm.

The morphological terminology follows Platnick and Shadab (1975) and Levy (2004). In the text we provide references only to the most useful publications, including books and revisions.

All specimens treated in this study are held in the following collections: CP – personal collection of A.V. Ponomarev (Rostov-on-Don, Russia); EMZ – personal collection of E.M. Zhukovets (Minsk, Belarus); ISEA – Siberian Zoological Museum, Institute for Systematics and Ecology of Animals, Novosibirsk, Russia, G.N. Azarkina; TNU – Zoology Department, V.I. Vernadsky Taurida National University, Simferopol, Ukraine (M.M. Kovblyuk); ZMMU – Zoological Museum of the Moscow State University, Moscow, Russia, K.G. Mikhailov; ZMT – Zoological Museum, University of Turku, Finland (S. Koponen); YMC – Yuri M. Marusik’s temporary collection in the Zoological Museum, University of Turku, Finland.

The following abbreviations are used in the text: AM, AL, PM, PL – anterior median, anterior lateral, posterior median and posterior lateral eyes; RTA – retrolateral tibial apophysis.

## Key to *Haplodrassus* species found in Crimea

### Males

**Table:** 

1	Terminal apophysis toothed (Fig. 37)	*Haplodrassus kulczynskii*
–	Terminal apophysis with 1–2 or without teeth	2
2	Total length ≤ 4 mm, carapace length ≤ 2 mm	*Haplodrassus minor*
–	Total length more than 4 mm, carapace longer than 2 mm	3
3	Terminal apophysis with plate-like bulge (Fig. 81)	*Haplodrassus umbratilis*
–	Terminal apophysis without plate-like bulge	4
4	Terminal apophysis with 1–2 teeth (Figs 23, 26)	5
–	Terminal apophysis without teeth (Figs 2, 5, 69, 72)	6
5	Terminal apophysis with 1 tooth, embolus without tooth (Fig. 26), metatarsus I with 2 ventral spines	*Haplodrassus isaevi*
–	Terminal apophysis with 2 teeth, embolus with tooth (Fig. 23), metatarsus I without ventral spines	*Haplodrassus dalmatensi*s
6	RTA with dorsal “step”-like keel (Figs 1, 4), embolus without tooth (Figs 2, 5)	*Haplodrassus bohemicus*
–	RTA without “step”-like keel (Figs 68, 71), embolus with tooth (Figs 69, 72)	7
7	Terminal apophysis short (Fig. 69), length/width ratio ca 2	*Haplodrassus pseudosignifer*
–	Terminal apophysis long (Fig. 72), length/width ratio ca 3	*Haplodrassus signifer*

### Females

**Table:** 

1	Epigynal pockets with long protrusion directed anteriorly (Fig. 38)	*Haplodrassus kulczynskii*
–	Epigynal pockets without long protrusion	2
2	Body length ≤ 5 mm, carapace ≤ 2 mm	*Haplodrassus minor*
–	Body longer than 5 mm, carapace longer than 2 mm	2
3	Epigynal fovea constricted anteriorly (Figs 28, 30)	4
–	Epigynal fovea not constricted anteriorly	5
4	Epigynal fovea with septum, fovea wider than spermathecae (Fig. 28), metatarsus IV with 3 retrolateral spines	*Haplodrassus dalmatensi*s
–	Epigynal fovea without septum, fovea narrower than spermathecae (Fig. 30), metatarsus IV with 4–5 retrolateral spines	*Haplodrassus isaevi*
5	Spermathecae oval (Figs 82–83)	*Haplodrassus umbratilis*
–	Spermathecae globular	6
6	Fovea without longitudinal groove (Figs 10, 12)	*Haplodrassus bohemicus*
–	Fovea with long longitudinal groove (Figs 75–78)	7
7	Fovea rectangular (longer than wide) (Fig. 75)	*Haplodrassus pseudosignifer*
–	Fovea square-shaped (length subequal to width) (Fig. 77)	*Haplodrassus signifer*

## Survey of species

### Haplodrassus

Chamberlin, 1922

http://species-id.net/wiki/Haplodrassus

#### Type species:

*Drassus hiemalis* Emerton, 1909.

#### Diagnosis.

Male palp with large terminal apophysis, thick embolus, hooked median apophysis and RTA flattened, often shifted dorsally. Epigyne with thick sclerotized lateral pockets and with one anterior hood. Posterior median eyes close together, separated by their radius or less (Platnick and Shadab 1975; Levy 2004). *Haplodrassus* is most related to *Orodrassus* Chamberlin, 1922 with three species from the Nearctic, but differs by having a flattened RTA (bifid or laterally expanded in *Orodrassus*), lacking a median epigynal projection (present in *Orodrassus*) and by the presence of lateral epigynal sclerites (absent in *Orodrassus*) (Platnick and Shadab 1975).

In terms of habitus and coloration *Haplodrassus* resembles only two other genera that occur in Crimea and the eastern Mediterranean: *Parasyrisca* Schenkel, 1963 and, to a lesser extent, *Drassodes* Westring, 1851. However, *Haplodrassus* is easily distinguished by having a large terminal apophysis (absent in the other genera), a flat retrolateral tibial apophysis widened dorsally (conical or flat and tapering in *Drassodes* and *Parasyrisca*), a broad embolus (cylindrical or hidden in the other genera), and the presence of heavily sclerotized lateral epigynal pockets (absent in *Drassodes* and *Parasyrisca*).

#### Distribution.

Holarctic and India (Platnick 2012).

### Haplodrassus bohemicus

Miller & Buchar, 1977

http://species-id.net/wiki/Haplodrassus_bohemicus

Figs 1–6 10–12

Haplodrassus bohemicus Miller and Buchar 1977: 163, pl. II, f. 1–6 (♂♀).
Haplodrassus bohemicus : Stefanovska et al. 2008: 37, f. 10–15 (♂♀).

#### Material.

UKRAINE, CRIMEA: Saky Distr.: 12 ♂♂, 2 ♀♀ (TNU), near Pribrezhnaya railway station, 30.04.-24.06.2000, M.M. Kovblyuk.

#### Additional material.

UKRAINE. Nikolaev Area: 6 ♂♂ (TNU), Pervomaysky Distr., Kuripchane Vil., 5.05.–8.06.2006, N.Yu. Polchaninova. Kherson Area: 2 ♂♂, 2 ♀♀ (TNU), Henichesk Distr., Arabatskaya strelka, 4 km S Henichesk Town, 23.05.–10.06.2011, N.A. Stasyuk. Donetsk Area: 4 ♂♂ (TNU), Pershotravnevy Distr., Belosaraiskaya Kosa, 11–22.06.2001, E.V. Prokopenko; 2 ♂♂ (TNU), Novoazovsk Distr., Khomutovo Vil., “Khomutovskaya Steppe”, N47°16', E38°10', 15–20.06.2004, N.Yu. Polchaninova; 1 ♂ (TNU), Slavyansky Distr., Svyatogorsk Town, N49°02', E37°39', 8–30.06.2004, N.Yu. Polchaninova. RUSSIA. Rostov Area: 7 ♂♂, 1 ♀ (TNU), Ust’-Donetsk Distr., near Razdorskaya Vil., «Pukhlyakovskye sklony», 10.05.–28.06.2004, A.V. Ponomarev.

#### Comparative material.

*Haplodrassus pugnans* (Simon, 1880): RUSSIA, Magadan Area: 1 ♂, 3 ♀♀ (ISEA, БИ-930), Ten’kynskyi Distr., env. Sibit-Tyellakh, Aborigen Field Station, *Betula*, 12–22.06.1983, A.V. Avershin.

#### Diagnosis.

*Haplodrassus bohemicus* is most similar to *Haplodrassus pugnans* (Simon, 1880), *Haplodrassus signifer* (C.L. Koch, 1839) and *Haplodrassus pseudosignifer* Marusiket al. 1996, but differs: 1) by the shape of RTA having a “step”-like dorsal margin (RTA dorsal margin without “step” in *Haplodrassus pugnans*, *Haplodrassus signifer* and *Haplodrassus pseudosignifer*); 2) by the apically directed embolus lacking a tooth (retrolaterally directed embolus with a tooth in *Haplodrassus pugnans*, *Haplodrassus signifer* and *Haplodrassus pseudosignifer*); 3) by the outlines of epigynal sclerites and relative proportions of the epigynal fovea (cf. Figs 10, 12 and 13, 75, 78).

#### Description.

Male measurements (n = 5). Total length 5.8–7.5 (6.7); carapace 2.7–3.2 (3.0) long, 2.0–2.6 (2.3) wide. Diameters of eyes and distances between them: AM 0.10–0.15 (0.12), AL 0.10–0.16 (0.13), PM 0.15–0.22 (0.19), PL 0.10–0.14 (0.12), AM-AM 0.09–0.12 (0.10), AM-AL 0.03–0.04 (0.04), PM-PM 0.03–0.04 (0.03), PM-PL 0.12–0.16 (0.14), AM-PM 0.12–0.18 (0.15), AL-PL 0.10–0.16 (0.14). Distances between anterior eyes and margin of clypeus: AM-clypeus 0.18–0.22 (0.20), AL-clypeus 0.10–0.18 (0.15).

Length of leg segments (male):

**Table:** 

**Leg**	**Femur**	**Patella**	**Tibia**	**Metatarsus**	**Tarsus**	**Total**
I	1.9–2.2 (2.1)	1.1–1.4 (1.2)	1.6–1.7 (1.6)	1.2–1.4 (1.3)	0.9–1.0 (1.0)	6.8–7.6 (7.2)
II	1.6–1.9 (1.8)	1.0–1.2 (1.1)	1.2–1.4 (1.3)	1.1–1.2 (1.2)	0.9–1.0 (1.0)	5.8–6.8 (6.3)
III	1.5–1.8 (1.6)	0.8–1.0 (0.9)	0.9–1.0 (1.0)	1.2–1.4 (1.3)	0.8–1.0 (0.9)	5.2–6.0 (5.6)
IV	2.1–2.3 (2.2)	1.0–1.2 (1.1)	1.6–1.7 (1.6)	1.8–2.0 (1.9)	1.0–1.2 (1.1)	7.5–8.3 (8.0)

Length of palp segments: femur 1.0–1.2 (1.1), patella 0.4–0.5 (0.5), tibia 0.3–0.5 (0.4), tarsus 1.0–1.2 (1.0). Cheliceral teeth: anterior – 2 (little; proximal tooth connected with keel of cheliceral groove), posterior – 2. Abdomen 2.9–4.0 (3.6) long, 1.8–2.2 (2.0) wide. Scutum is absent. Basal segment of anterior (inferior) spinnerets 0.5–0.6 (0.6) long. Coloration light brown, as in most *Haplodrassus* species.

**Figures 1–9.**
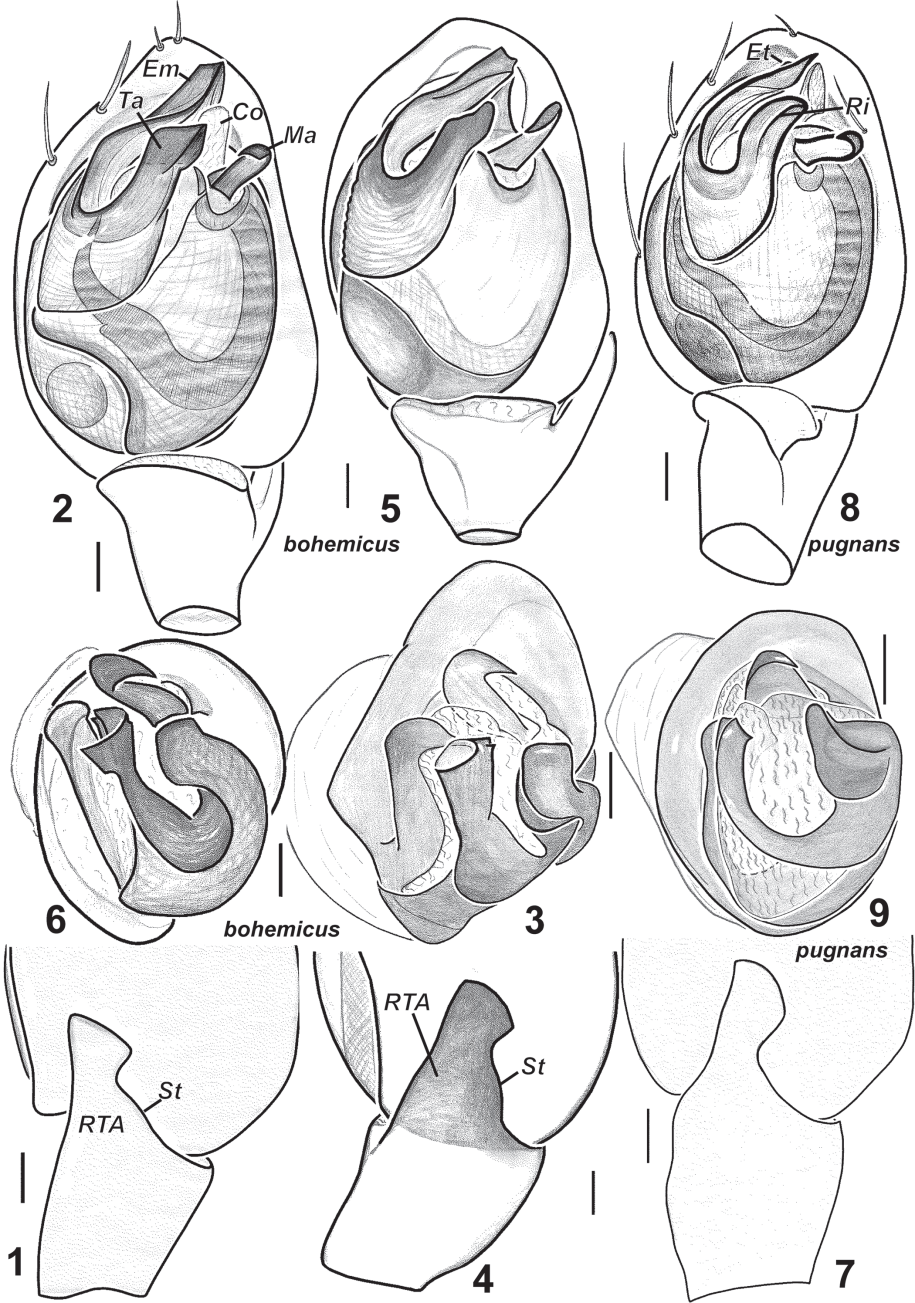
Males of *Haplodrassus bohemicus* (**1–3** from Rostov Area, **4–6** from Crimea) and *Haplodrassus pugnans* (**7–9** from Magadan Area): **1, 4, 7** RTA, retrolateral view **2, 5, 8** palp, ventral view **3, 6, 9** bulbus, apical view. Abbreviations: ***Co***conductor; ***Em***embolus; ***Et***tooth of embolus; ***Ma***median apophysis; ***Ri*** ridgeof terminal apophysis; ***RTA*** retrolateral tibial apophysis; ***St***“step”-like keel of RTA; ***Ta***terminal apophysis.

Palp as in Figs 1–6. RTA with a “step” (*St*) on the promargin, terminal apophysis almost straight, without distinct ridge, embolus slightly twisted and lacks a tooth.

Female measurements (n = 2). Total length 7.8–8.0; carapace 2.7–3.5 long, 2.0–2.7 wide. Abdomen 4.3–5.1 long, 2.7–3.1 wide. Coloration light brown, as in most *Haplodrassus* species.

**Figures 10–14.**
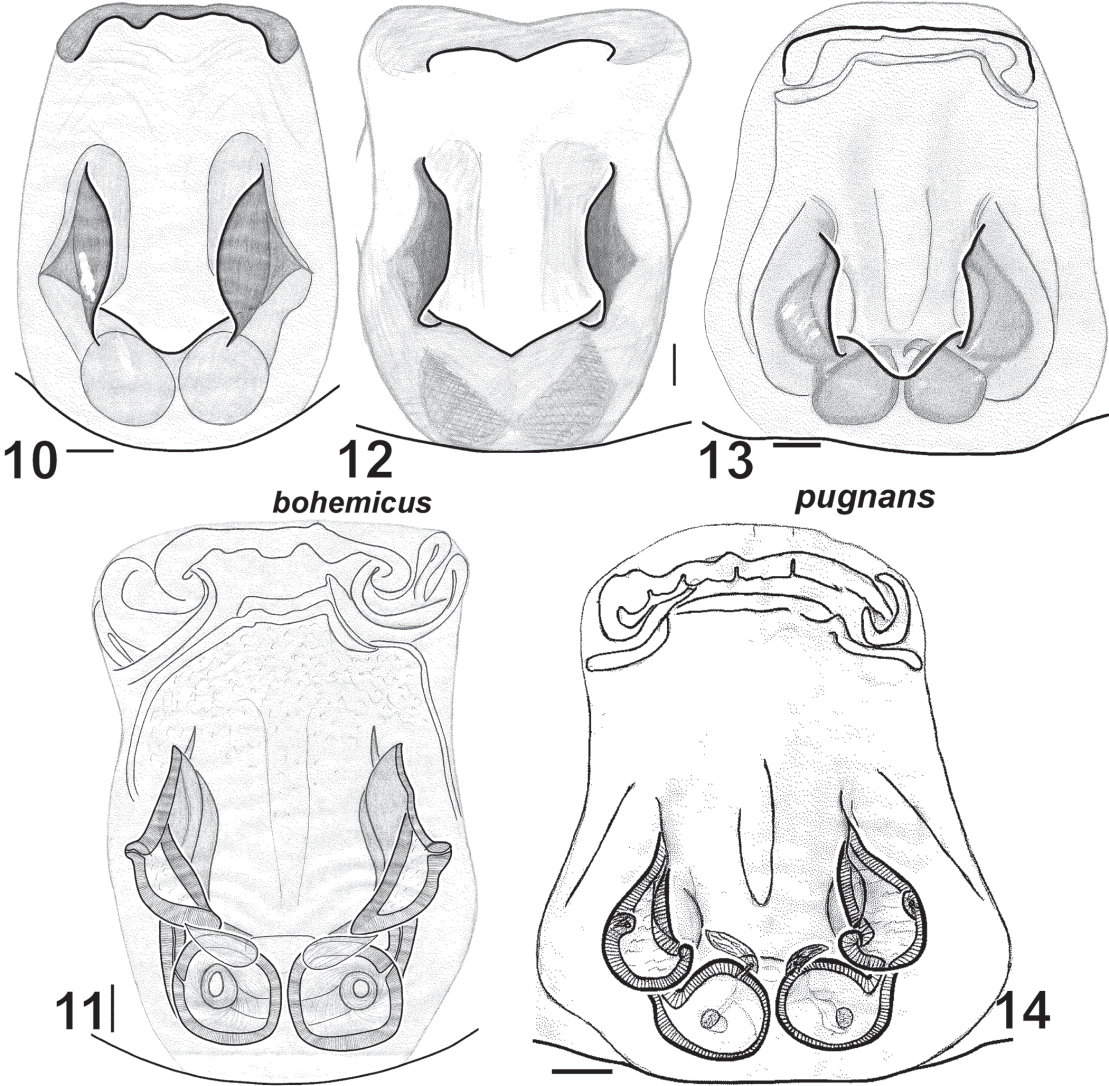
Females of *Haplodrassus bohemicus* (**10–11** from Rostov Area, **12** from Crimea) and *Haplodrassus pugnans* (**13–14** from Magadan Area): **10, 12–13** epigyne, ventral view **11, 14** epigyne, dorsal view.

Epigyne as in Figs 10–12. Lateral pockets long, sub-parallel, spermathecae globular. Fovea of epigyne without narrow longitudinal groove.

#### Distribution.

Czech Republic, Macedonia, Greece, Ukraine (Nikolaev, Kherson, Donetsk Areas, Crimea), Russia (Rostov and Stavropol Areas, Kalmykya, Dagestan) (Miller and Buchar 1977; Ponomarev and Tsvetkov 2006; Stefanovska et al. 2008; Ponomarev et al. 2011; present data).

#### Comments.

*Haplodrassus bohemicus* is a new species record for the Crimean fauna.

#### Habitats.

Meadows, steppes and sand dunes.

#### Phenology.

♂♀ – V-VI. In Crimea the peak activity of adults occurs in May.

### Haplodrassus cognatus

(Westring, 1861)

http://species-id.net/wiki/Haplodrassus_cognatus

Figs 15–16

Haplodrassus cognatus : Tullgren 1946: 106, f. 31C, pl. 17, f. 221–224 (♂♀).
Haplodrassus cognatus : Miller and Buchar 1977: 168, pl. III, f. 8–10 (♂♀).
Haplodrassus cognatus : Grimm 1985: 134, f. 155, 168–169 (♂♀).
Haplodrassus cognatus : Roberts 1998: 109, f. (♂♀).
Haplodrassus cognatus : Almquist 2006: 407, f. 351a–f (♂♀).
Haplodrassus cognatus : Kamura 2007: 97, f. 7–8 (♂).
Haplodrassus cognatus : Kamura 2009: 485, f. 32–34 (♂♀). For a complete list of references see Platnick (2012).

#### Records from Crimea.

Bragina (1984); Kovblyuk (2004a,b, 2006); Kovblyuk et al. (2008).

#### Note.

The earlier record of *Haplodrassus cognatus* from Crimea was based on specimens of unknown sex and number from Karadag Nature Reserve (Bragina 1984). *Haplodrassus cognatus* is absent in our material from Crimea, although we have large collections, especially from the Karadag Reserve). It is reasonable to conclude that the earlier records of *Haplodrassus cognatus* from Crimea represent a misidentified material.

#### Additional material.

UKRAINE. Donetsk Area: 1 ♀ (TNU), Slavyansky Distr., Svyatogorsk Town, N49°02', E37°39', *Quercus*, 7.06–9.07.2005, N.Yu. Polchaninova.

#### Comparative material.

*Haplodrassus silvestris* (Blackwall, 1833): UKRAINE. Chernovtsy Area: 1 ♂ (TNU № 2153), Tsetsyno Town, *Fagus* wood, 23.04–18.05.2009, V.V. Garashchuk & T.O. Auzyak. Kharkiv Area: 1 ♂, 1 ♀ (TNU), Veliko-Burlukskiy Distr., Nesterivka Vill., N49°53', E37°17', 14.06–14.07.2003, N.Yu. Polchaninova. RUSSIA. Belgorod Area: 1 ♀ (TNU), Borisobsky Distr., Borisovka Town, “Les na Vorkle” Reserve, N50°38', E35°58', 5.07.unknown year, N.Yu. Polchaninova.

#### Diagnosis.

*Haplodrassus cognatus* can be distinguished from all other *Haplodrassus* species by its straight terminal apophysis with a basal tooth in males, and by the shape of the fovea and wide anterior hood (*Ah*) in females.

**Figures 15–21.**
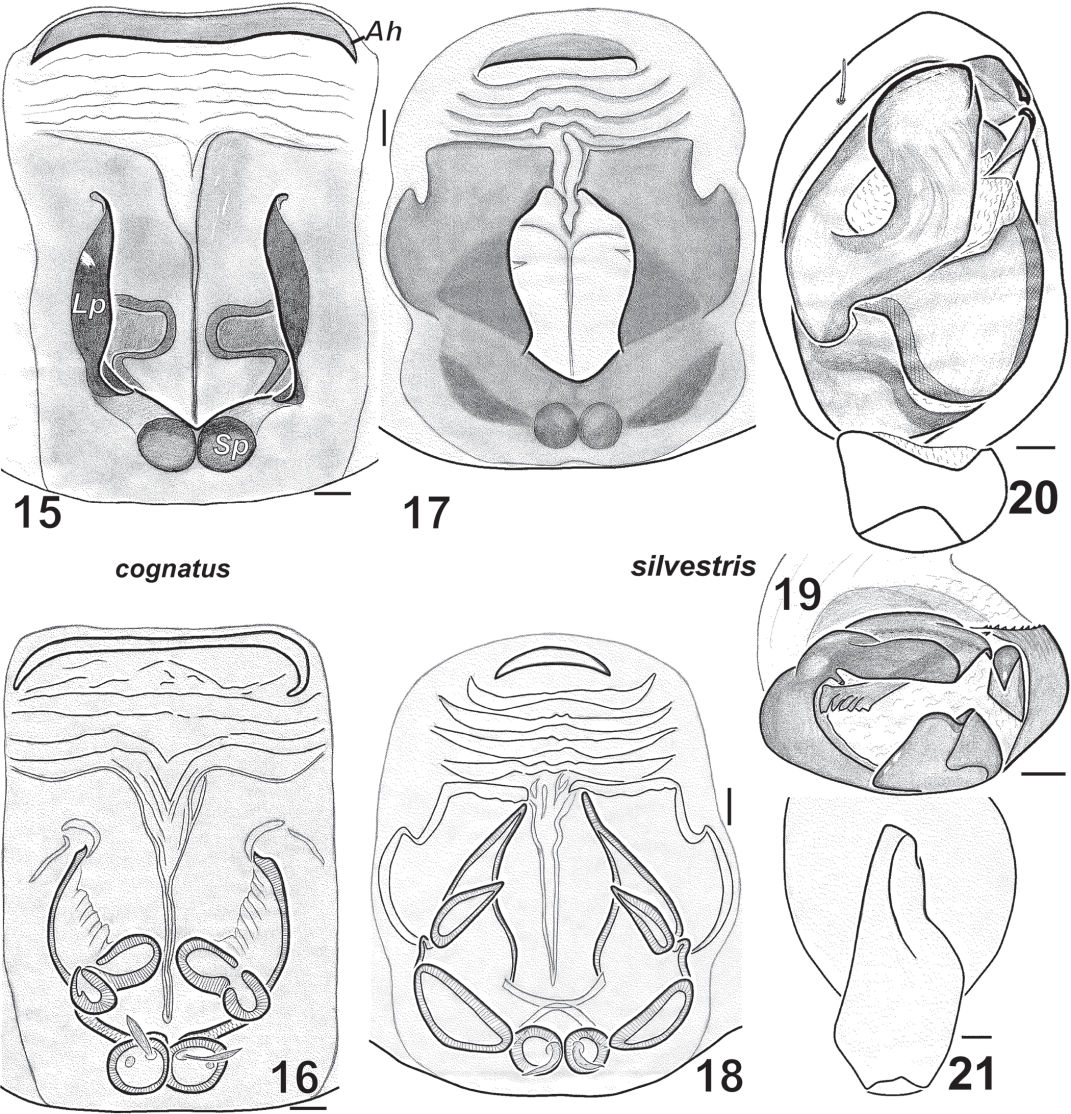
*Haplodrassus cognatus* (**15–16** from Donetsk Area)and *Haplodrassus silvestris* (**17–21** from Kharkiv Area): **15, 17** epigyne, ventral view **16, 18** epigyne, dorsal view **19** bulbus, apical view **20** palp, ventral view **21** RTA, retrolateral view. Abbreviations: ***Ah***anterior hood of epigyne; ***Lp*** lateral pocket of epigyne; ***Sp*** spermatheca.

#### Distribution.

It has a trans-Palearctic boreo-nemoral range and occurs from France to Hokkaido, north to north Ural and Tomsk, and south to Greece (Marusik et al. 2000; Helsdingen 2010; Platnick 2012).

#### Phenology.

In Central Europe ♂♀ – III-X (Nentwig et al. 2011).

### Haplodrassus dalmatensis

(L. Koch, 1866)

http://species-id.net/wiki/Haplodrassus_dalmatensis

Figs 22–24 28–29

Haplodrassus dalmatensis : Tullgren 1946: 100, pl. 16, f. 201–203 (♂♀).
Haplodrassus dalmatensis : Miller and Buchar 1977: 170, pl. IV, f. 1–3 (♂♀).
Haplodrassus dalmatensis : Grimm 1985: 138, f. 156, 164–165 (♂♀).
Haplodrassus dalmatensis : Roberts 1985: 66, f. 24a (♂♀).
Haplodrassus dalmatensis : Roberts 1998: 110, f. (♂♀).
Haplodrassus dalmatensis : Levy 2004: 23, f. 57–61 (♂♀).
Haplodrassus dalmatensis : Almquist 2006: 408, f. 352a–e (♂♀). For a complete list of references see Platnick (2012).

#### Records from Crimea.

Apostolov and Onchurov (1998); Onchurov (1998); Mikhailov (2000); Kovblyuk (2004a,b, 2006); Kovblyuk et al. (2008).

#### Material.

UKRAINE, CRIMEA:Bakhchisaray Distr.: 1 ♂ (TNU), Crimean State Nature Reserve, kordon Asport, 29.06.2001, M.M. Kovblyuk. Feodosiya Distr.: 4 ♂♂, 4 ♀♀ (TNU), Karadag Nature Reserve, 25.05.2003–21.11.2008, M.M. Kovblyuk, O.V. Kukushkin, A.A. Nadolny. Saky Distr.: 5 ♂♂, 7 ♀♀ (TNU), near Pribrezhnaya railway station, 19.05.–3.07.2000, M.M. Kovblyuk. Sevastopol Distr.: 2 ♂ (EMZ), Khersones, 29.05.1996 & 19.06.1998, M.M. Kovblyuk. Simferopol Distr.: 1 ♂, 1 ♀ (EMZ), near Simferopol water reservoir, 30.05.1996, M.M. Kovblyuk; 1 ♀ (TNU), near Fersmanovo Vill., ~ 250 m, 23.06.–16.07.2000, M.M. Kovblyuk; 1 ♀ (TNU), Chatyr-Dagh, Orlinoe canyon, 10–25.06.2000, M.M. Kovblyuk; 3 ♂♂, 1 ♀ (TNU), near Skvortsovo Vill., 19.05.–10.07.2002, M.M. Kovblyuk; 1 ♀ (TNU), Krasnolesye Vill., 10.07.2002, Ya.I. Ibragimova. Sudak Distr.: 2 ♂♂, 2 ♀♀ (TNU), 10 km W Sudak, Mezhdurechie Vill., 23.05–24.06.2010, M.K. Yusufova.

#### Additional material.

UKRAINE. Kherson Area: 1 ♂ (TNU), Henichesk Distr., Arabatskaya strelka, 4 km S Henichesk Town, 1–10.06.2010, N.A. Stasyuk; 1 ♀ (TNU), Arabatskaya strelka, 7 km S Henichesk Town, 6.07.2010, N.A. Stasyuk.

#### Diagnosis.

*Haplodrassus dalmatensis* can be easily distinguished from all other congeners by the shape of the terminal apophysis with two tooth-like apical processes and by the strong tooth on the embolus in males, and also by the shape of the epigynal fovea with a peculiar medial septum and converging lateral pockets in females.

**Figures 22–27.**
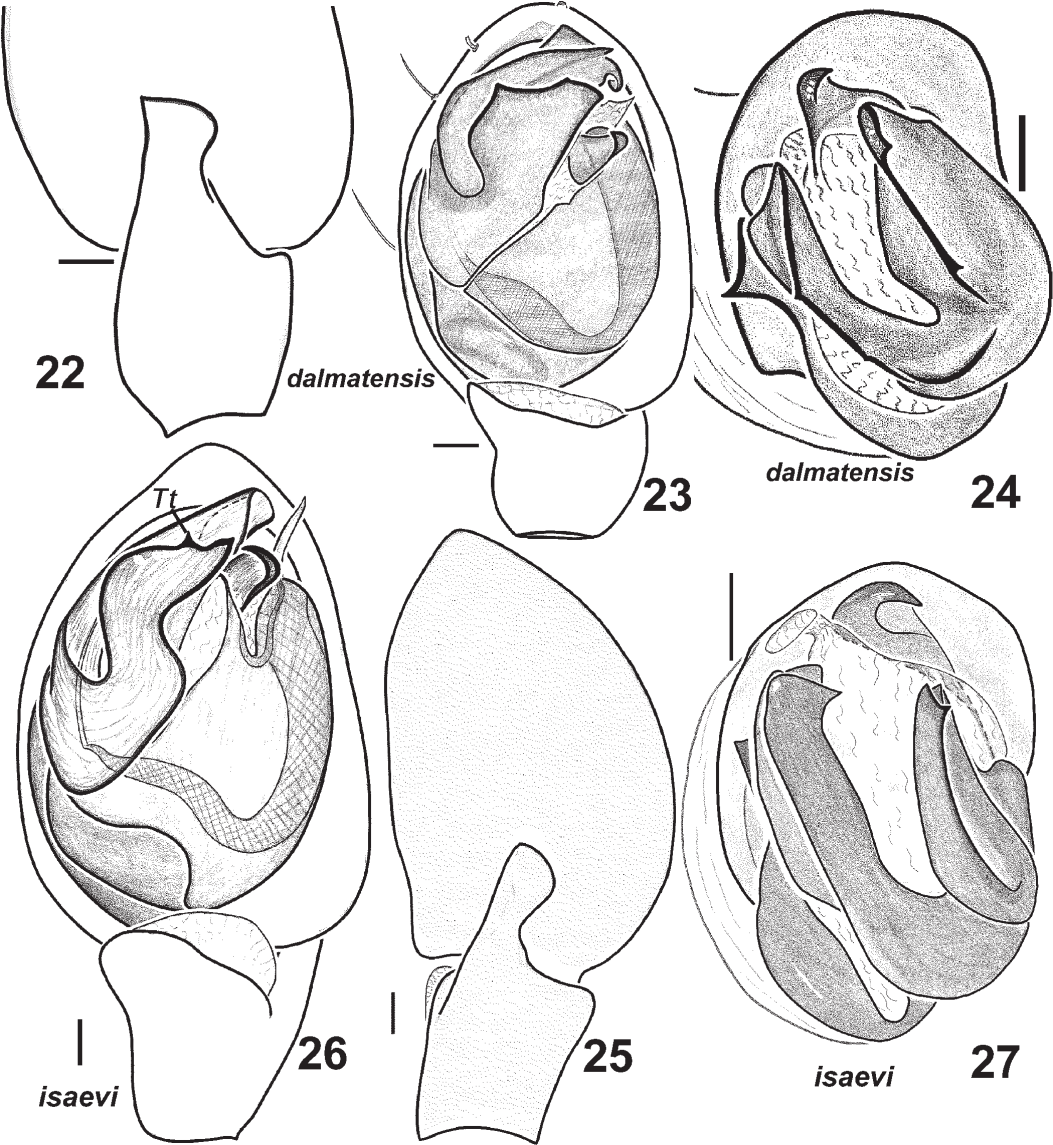
Males of *Haplodrassus dalmatensis* (**22–24** from Crimea) and *Haplodrassus isaevi* (**25–26** paratype from Rostov Area, **27** from Crimea): **22, 25** RTA, retrolateral view **23, 26** palp, ventral view **24, 27** bulbus, apical view. Abbreviations: ***Tt*** tooth-like process of terminal apophysis.

**Figures 28–31.**
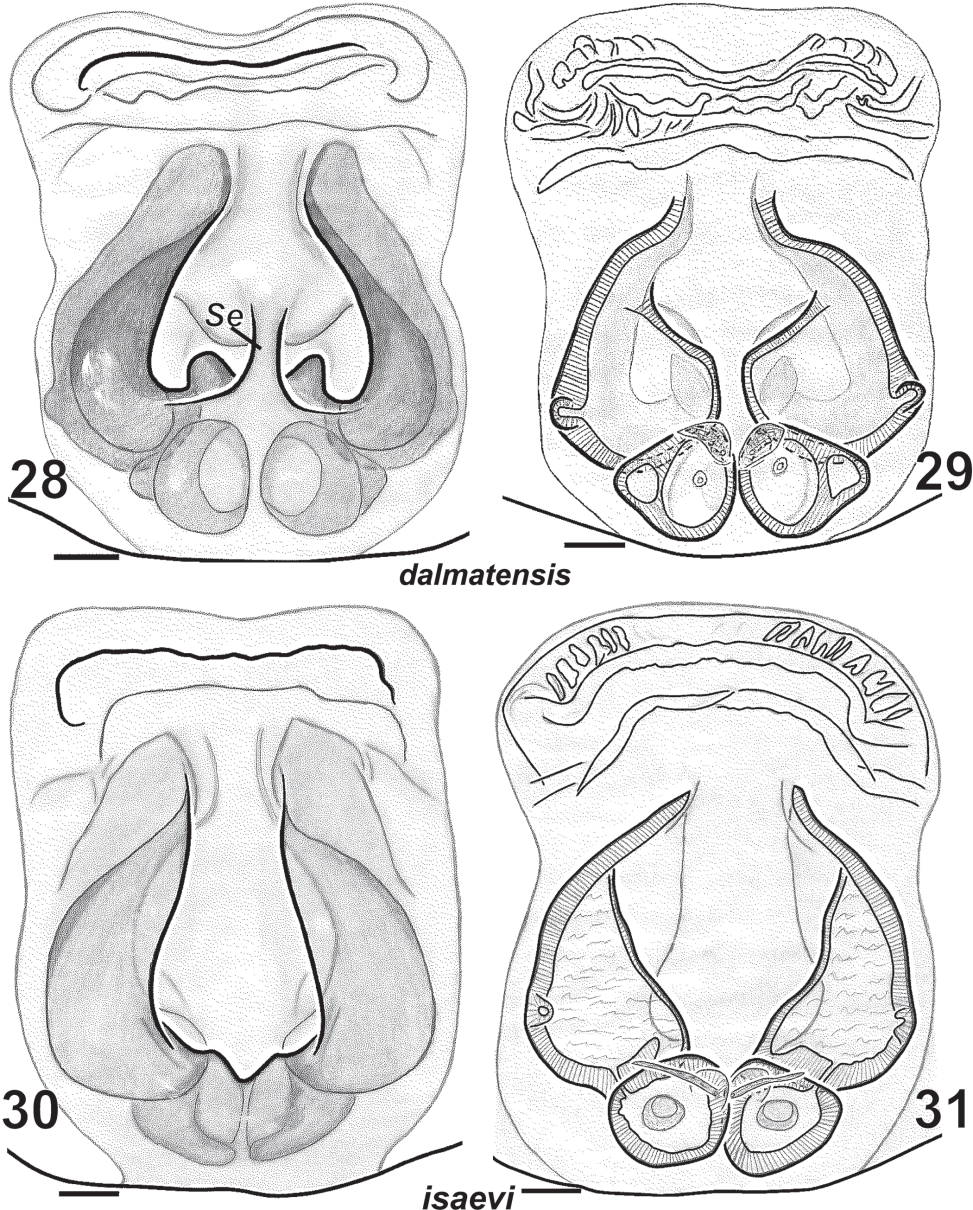
Females of *Haplodrassus dalmatensis* (**28–29** from Crimea) and *Haplodrassus isaevi* (**30–31** from Crimea): **28, 30** epigyne, ventral view **29, 31** epigyne, dorsal view. Abbreviations: ***Se***septum.

#### Distribution.

West and Central Palaearctic: North Africa, Europe, South Urals, Caucasus, Anatolia, Near East, Kazakhstan, Western Turkmenistan and mountains of South Siberia (Mikhailov 2000; Tuneva and Esyunin 2003; Levy 2004; Helsdingen 2010).

#### Habitats.

Juniper forests, forest strips (=shelterbelts), grasslands, steppes, meadows, salt marshes.

#### Phenology.

In Crimea ♂♀ – V-VI, ♀♀ – VII, XI-XII, the peak of activity in adults occurs in June. In Britain, the peak is in June (Harvey et al. 2002), as in Crimea. In Central Europe ♂♀ – IV-VII (Nentwig et al. 2011). In Israel the phenology is very different: ♂♀ – I-IV, ♂♂ – XII, ♀♀ – V-VII (Levy 2004).

### Haplodrassus invalidus

(O. P.-Cambridge, 1872)

http://species-id.net/wiki/Haplodrassus_invalidus

Figs 32–34

Drassus invalidus O. P.-Cambridge, 1872a: 237, pl. 15, f. 14 (♂).
Haplodrassus vignai Di Franco, 1996: 173, f. 1–4 (♂♀).
Haplodrassus invalidus : Levy 2004: 31, f. 70–73 (♂♀). For a complete list of references see Platnick (2012).

#### Material.

AZERBAIJAN. 1 ♂ (TNU), Gobustan, Beyuk-Dash, 17.05.2001, E.F. Huseynov.

#### Diagnosis.

**Figures 32–34.**
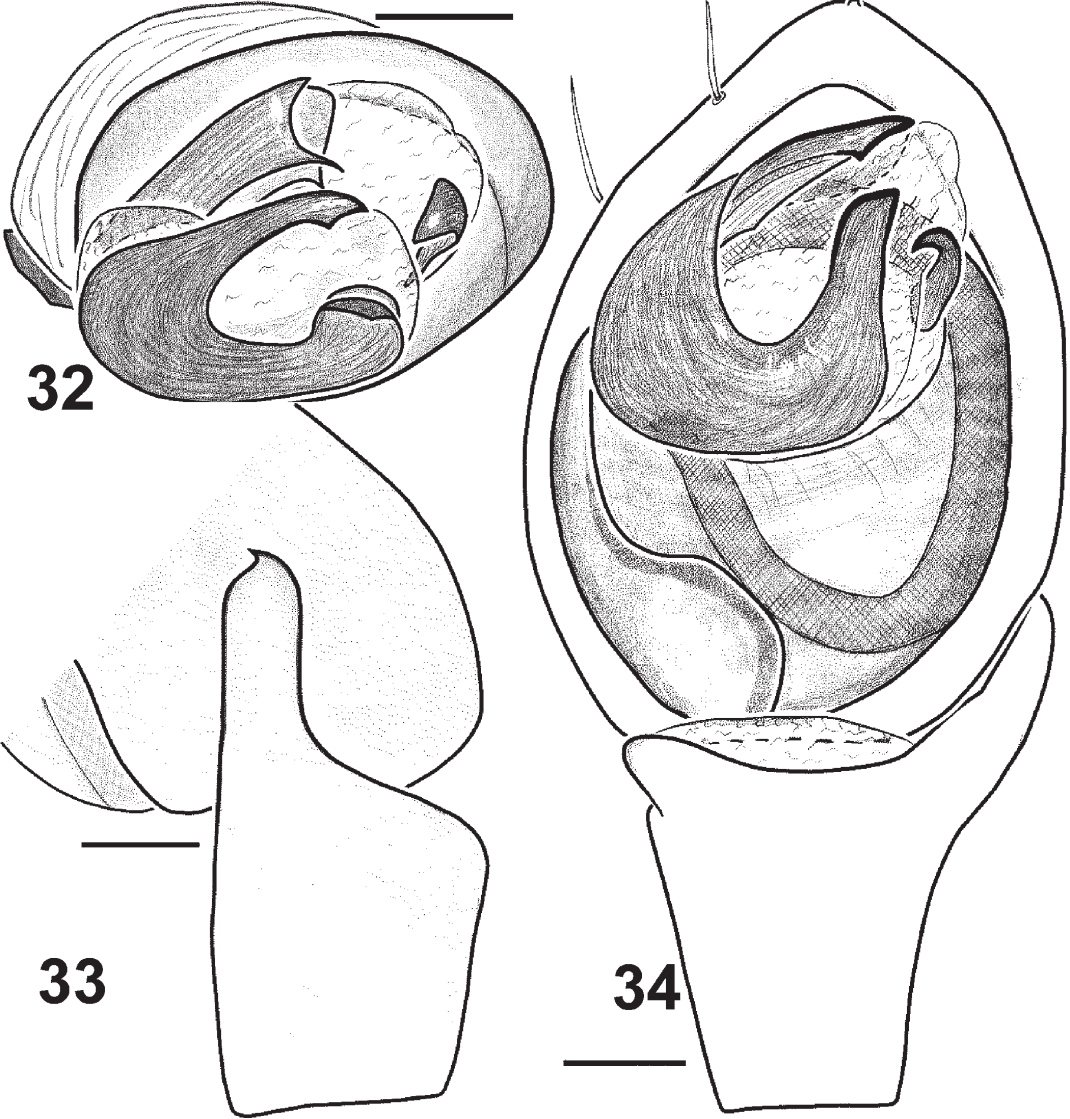
Males of *Haplodrassus invalidus* from Azerbaijan: **32** bulbus, apical view **33** RTA, retrolateral view **34** palp, ventral view.

Males of *Haplodrassus invalidus* can be distinguished from all other *Haplodrassus* species by the peculiar thin embolus with an inner spur-like process, and also the peculiar shape of the RTA, which is not indented and has a claw-like tip (Figs 32–34).

#### Description.

Well described by Levy (2004).

#### Distribution.

Spain, Corsica, Italy (mainland and Sicily), Turkey, Israel and Azerbaijan (Levy 2004; Platnick 2012; present data).

#### Comments.

*Haplodrassus invalidus* is a new species record for the fauna of Azerbaijan, Caucasus and the former Soviet Union. Azerbaijan is the easternmost point of the known distribution range.

### Haplodrassus isaevi

Ponomarev & Tsvetkov, 2006

http://species-id.net/wiki/Haplodrassus_isaevi

Figs 25–27 30–31

Haplodrassus isaevi Ponomarev & Tsvetkov, 2006: 9, f. 12–14 (♂♀).
Haplodrassus isaevi : Piterkina and Ovtsharenko 2007: 1426, f. 1.1–6 (♂♀).

#### Records from Crimea.

Kovblyuk et al. (2008);Kovblyuk et al. (2009).

#### Type material.

RUSSIA, ROSTOV AREA: 3 ♂♂ paratypes (TNU from CP 18.24.8), Orlovskyi Distr., Rostov Reserve, 6.10.2002, A.V. Ponomarev.

#### Material.

UKRAINE, CRIMEA: Sudak Distr.: 2 ♂♂, 3 ♀♀ (TNU), 10 km W Sudak, Mezhdurechie Vill., 3.10.–7.11.2010, A.K. Yusufova. Feodosiya Distr.: 21 ♂♂, 10 ♀♀ (TNU), Karadag Nature Reserve, 28.05.2003–19.12.2008, M.M. Kovblyuk, O.V. Kukushkin.

#### Diagnosis.

*Haplodrassus isaevi* is most similar to *Haplodrassus dalmatensis* but differs by the shape of the terminal apophysis, which has only one tooth-like process (two tooth-like apical processes in *Haplodrassus dalmatensis*), in lacking a tooth on the embolus (embolic tooth present in *Haplodrassus dalmatensis*), and also by the proportions of the epigyne. Differences also occur in the spination of certain leg segments: male metatarsus I with two ventral spines in *Haplodrassus isaevi*, but without spines in *Haplodrassus dalmatensis*; female metatarsus IV with 4–5 retrolateral spines in *Haplodrassus isaevi*, but with 3 spines in *Haplodrassus dalmatensis*.

#### Description.

Males (n =5) and females (n = 5). Measurements (♂ / ♀): total length 5.4–7.2 (6.4) / 5.5–7.4 (6.3); carapace 2.3–2.9 (2.7) / 2.4–3.0 (2.7) long, 1.9–2.4 (2.2) / 1.9–2.2 (2.0) wide; abdomen 3.1–4.3 (3.7) / 3.0–4.4 (3.7) long, 1.7–2.3 (2.0) / 1.8–2.8 (2.3) wide.

Length of leg segments:

**Table:** 

**Leg**	**Femur**	**Patella**	**Tibia**	**Metatarsus**	**Tarsus**	**Total**
I♂	1.6–2.0 (1.8)	1.1–1.3 (1.2)	1.3–1.6 (1.4)	1.0–1.2 (1.1)	0.8–1.0 (1.0)	5.8–7.1 (6.6)
II♂	1.4–1.7 (1.6)	0.9–1.2 (1.1)	1.0–1.3 (1.2)	0.8–1.1 (1.0)	0.8–0.9 (0.9)	5.0–6.3 (5.8)
III♂	1.3–1.6 (1.5)	0.7–0.9 (0.8)	0.8–1.0 (0.9)	0.9–1.2 (1.1)	0.6–0.8 (0.7)	4.3–5.4 (5.0)
IV♂	1.7–2.0 (1.9)	1.0–1.2 (1.3)	1.3–1.6 (1.5)	1.4–1.8 (1.6)	0.8–1.0 (0.9)	6.3–7.6 (7.0)
I♀	1.4–1.8 (1.7)	0.9–1.2 (1.1)	1.0–1.4 (1.3)	0.8–1.2 (1.0)	0.8–0.9 (0.8)	4.9–6.6 (5.9)
II♀	1.4–1.7 (1.5)	0.9–1.0 (1.0)	1.0–1.2 (1.1)	0.8–1.1 (0.9)	0.7–0.9 (0.8)	5.0–5.9 (5.4)
III♀	1.2–1.4 (1.3)	0.7–0.8 (0.8)	0.7–0.9 (0.8)	0.9–1.1 (1.0)	0.6–0.8 (0.7)	4.2–5.2 (4.8)
IV♀	1.6–2.0 (1.8)	0.9–1.1 (1.0)	1.2–1.6 (1.4)	1.3–1.8 (1.5)	0.8–1.0 (0.9)	5.8–7.4 (6.6)

Length of palp segments (male / female): femur 1.0–1.2 (1.1) / 0.8–1.1 (1.0), patella 0.4–0.5 (0.4) / 0.4–0.5 (0.5), tibia 0.3–0.4 (0.3) / 0.3–0.4 (0.4), tarsus 1.0–1.2 (1.0) / 0.6–0.7 (0.7).

Chelicerae with 2–3 promarginal and 2 retromarginal teeth in males and females. Number of promarginal teeth varies from 3 (most common) to 2 (seldom). One female studied had one chelicera with 3 and the other chelicera with 2 promarginal teeth. Coloration grey.

Male palp as in Figs 25–27. Terminal apophysis sharply turned, without ridge, but with tooth (*Tt*) in subterminal part, embolus without tooth.

Epigyne as in Figs 30–31. Fovea long, lateral pockets slightly converging, foveal width less that spermathecal span.

#### Distribution.

Greece, Ukraine (Crimea), Russia (Rostov Area), Kazakhstan (West-Kazakhstan Area) (Ponomarev and Tsvetkov 2006; Piterkina and Ovtsharenko 2007; Platnick 2012; present data).

#### Habitats.

Steppes.

#### Phenology.

In Crimea ♂♀ – X-XII, ♀♀ – II-III, V, the peak activity of adults occurs in December.

#### Comments.

In Crimea we found both closely related species, *Haplodrassus dalmatensis* and *Haplodrassus isaevi*, to be syntopical in two localities (Sudak Distr., 10 km W Sudak, Mezhdurechie Vill. and Feodosiya Distr., Karadag Nature Reserve). However, these species have quite different phenologies and adults of the two different species do not co-occur. The reproductive period of *Haplodrassus dalmatensis* is in May-July with the peak in June, and in *Haplodrassus isaevi* adults can be found in October-December, with their peak of activity in December.

### Haplodrassus kulczynskii

Lohmander, 1942

http://species-id.net/wiki/Haplodrassus_kulczynskii

Figs 35–39

Haplodrassus kulczynskii : Miller and Buchar 1977: 170, pl. IV, f. 7–10 (♂♀).
Haplodrassus kulczynskii : Grimm 1985: 141, f. 152, 162–163 (♂♀).
Haplodrassus kulczynskii : Roberts 1998: 111, f. (♂♀).
Haplodrassus kulczynskii : Marusik et al. 2007: 43, f. 5–10 (♂♀). For a complete list of references see Platnick (2012).

#### Records from Crimea.

Kovblyuk (2006).

#### Material.

UKRAINE, CRIMEA:Simferopol Distr.: 2 ♂♂, 1 ♀ (YMC), near Fersmanovo Vill., ~ 250 m, 18.04.–1.05.2000, M.M. Kovblyuk; 1 ♂, 1 ♀ (YMC), Chatyr-Dag Mt., Orlinoe canyon, 27.04.–1.06.2000, M.M. Kovblyuk. Yalta Distr.:2 ♂♂ (TNU), Nikitskaya Yaila Mt. (=Skrinita), 22.04.–25.05.2001, M.M. Kovblyuk.

#### Additional material.

UKRAINE. Nikolaev Area: 1 ♂ (TNU), Pervomaysky Distr., Migiya Vill., 5.05.–8.06.2006, N.Yu. Polchaninova. AZERBAIJAN. Lenkoran Distr.: 1 ♀ (TNU), Alexeevka Vill., 27.04.2001, E.F. Huseynov.

#### Diagnosis.

*Haplodrassus kulczynskii* is similar to *Haplodrassus rugosus* Tuneva, 2005 from Kazakhstan and *Haplodrassus taepaikensis* Paik, 1992 from Korea and the Russian Far East. Both species have a toothed terminal apophysis. *Haplodrassus kulczynskii* can be easily distinguished from similar species by having a much wider terminal apophysis, having a step-like subterminal outgrowth on the dorsal margin of the RTA (in *Haplodrassus rugosus* and *Haplodrassus taepaikensis* such an outgrowth is absent), and by the longer lateral pockets of the epigyne (in *Haplodrassus taepaikensis* they are shorter; the female of *Haplodrassus rugosus* is unknown).

**Figures 35–39.**
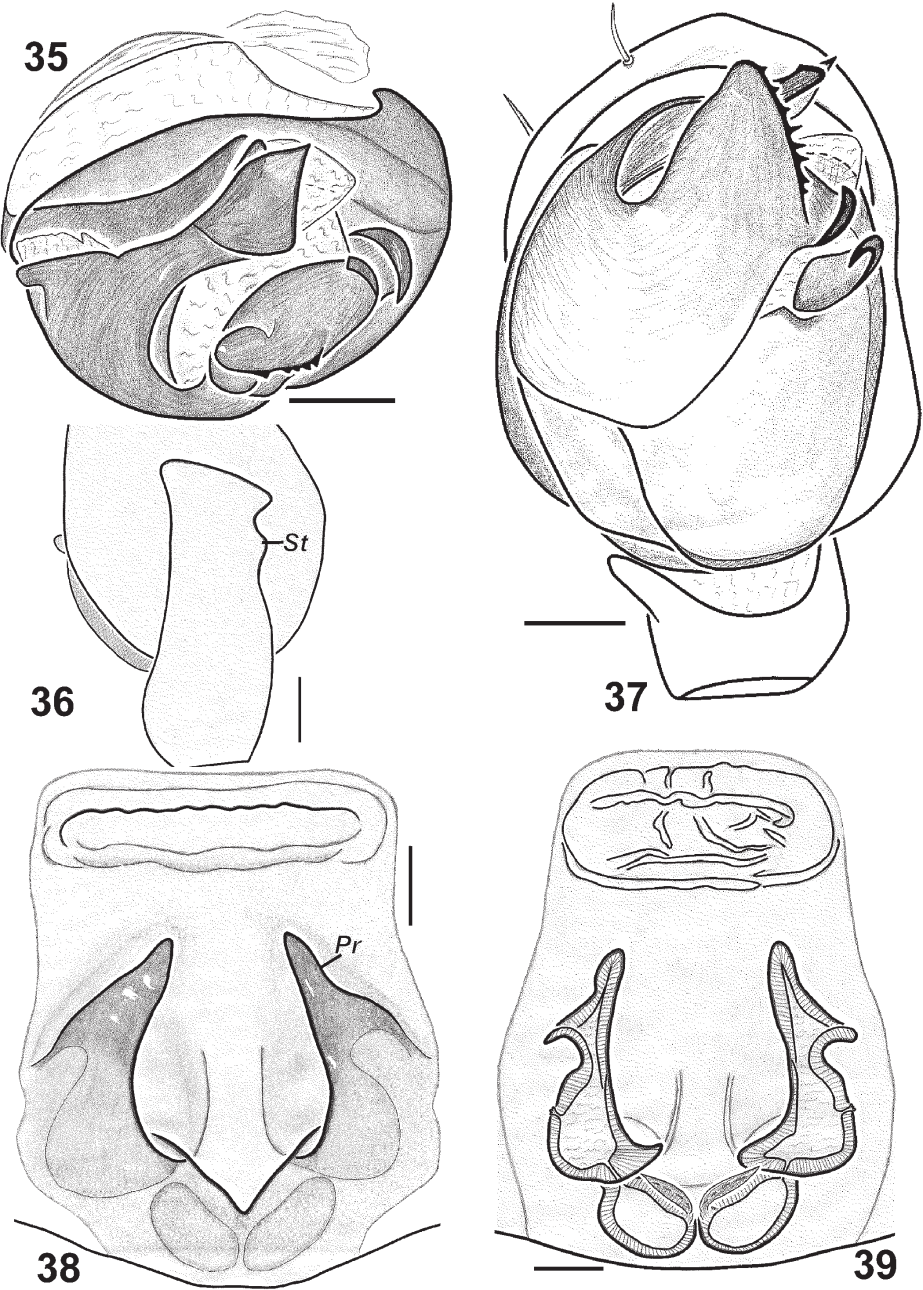
*Haplodrassus kulczynskii* from Crimea: **35** bulbus, apical view **36** RTA, retrolateral view **37** palp, ventral view **38** epigyne, ventral view **39** epigyne, dorsal view. Abbreviations: ***Pr*** protrusion of epigynal pocket; ***St*** “step”-like keel of RTA.

#### Distribution.

West Palaearctic – Far East disjunct nemoral-subtropical range: Central and Southern Europe to Urals, Caucasus, Turkey, Eastern China, Far East Russia and Korea (Mikhailov 1997; Tuneva and Esyunin 2003; Helsdingen 2010; Platnick 2012).

#### Habitats.

Steppe.

#### Phenology.

In Crimea ♂♀ – IV-V, the peak of activity in adults occurs in April. In Central Europe ♂♀ – IV-VIII (Nentwig et al. 2011).

### Haplodrassus minor

(O. P.-Cambridge, 1879)

http://species-id.net/wiki/Haplodrassus_minor

Figs 43–49 52 [Fig F10] –65

Haplodrassus minor : Miller and Buchar 1977: 170, pl. IV, f. 4–6 (♂).
Haplodrassus minor : Grimm 1985: 144, f. 176–178 (♂♀).
Haplodrassus minor : Roberts 1985: 66, f. 24c (♂♀).
Haplodrassus minor : Tuneva and Esyunin 2003: 232, f. 27–33 (♂♀). For a complete list of references see Platnick (2012).

#### Records from Crimea.

Kovblyuk (2006).

#### Material.

Ukraine. CRIMEA. Lenino Distr.: 1 ♀ (EMZ), Kerch peninsula, NW coast of Aktash lake, 8.06.1999, M.M. Kovblyuk. Saki Distr.: 5 ♂♂ (TNU), near Pribrezhnaya railway station, 9.05.–3.07.2000, M.M. Kovblyuk. Simferopol Distr.: 1 ♂ (TNU), Kirpichnoe Vill., 31.05.–12.06.1997, M.M. Kovblyuk; 18 ♂♂, 5 ♀♀ (YMC, TNU), Skvortsovo Vill., 27.04.–10.07.2002, M.M. Kovblyuk. Sovietsky Distr.: 1 ♂ (TNU), Uvarovka Vill., 28.04.1999, M.M. Kovblyuk. Yalta Distr.: 1 ♂ (TNU), Yalta, Nikita Vill., 13–30.05.2000, M.M. Kovblyuk.

#### Additional material.

UKRAINE. Donetsk Area: 1 ♂ (TNU), Slavyansk Distr., Dronovka Vil., 4–8.07.2002, E.V. Prokopenko; 1 ♂, 1 ♀ (TNU), Volodarsky Distr., Nazarovka Vil., “Kamennye Mogily” Nature Reserve, N47°20', E37°06', 20.06.1983, N.Yu. Polchaninova. Nikolaev Area: 1 ♂, 1 ♀ (TNU), Ochakov Distr., Pokrovka Vil., ‘Volyzhin Les’ Department of the Chernomorsky Nature Reserve, 21.05.1987, N.Yu. Polchaninova; 2 ♂♂ (TNU), Pervomaysky Distr., Kuripchane Vil., 6.05.-8.06.2006, N.Yu. Polchaninova. Drawings from these specimens – see in Figs 43–45, 52–53. RUSSIA. Orenburg Area: 5 ♂♂ (TNU), Kuvandyk Distr., Aituar Vill., 22.05.1996, 5.07.2002, N.S. Mazura, T.K. Tuneva; 4 ♂♂, 1 ♀ (TNU), Sol-Iletsk Distr., Chybynda, 5–12.06.2000, S.L. Esyunin. POLAND. 2 ♂♂, 2 ♀♀ (TNU), “Krusne hoty a Polsko, 2001–2002, leg. Prof. E. Kula”.

#### Comparative material. 

*Haplodrassus deserticola* Schmidt & Krause, 1996 from the Canary Islands: 2 ♂♂, 1 ♀ (TNU), leg. et det. J. Wunderlich.

#### Diagnosis. 

**Figures 40–49.**
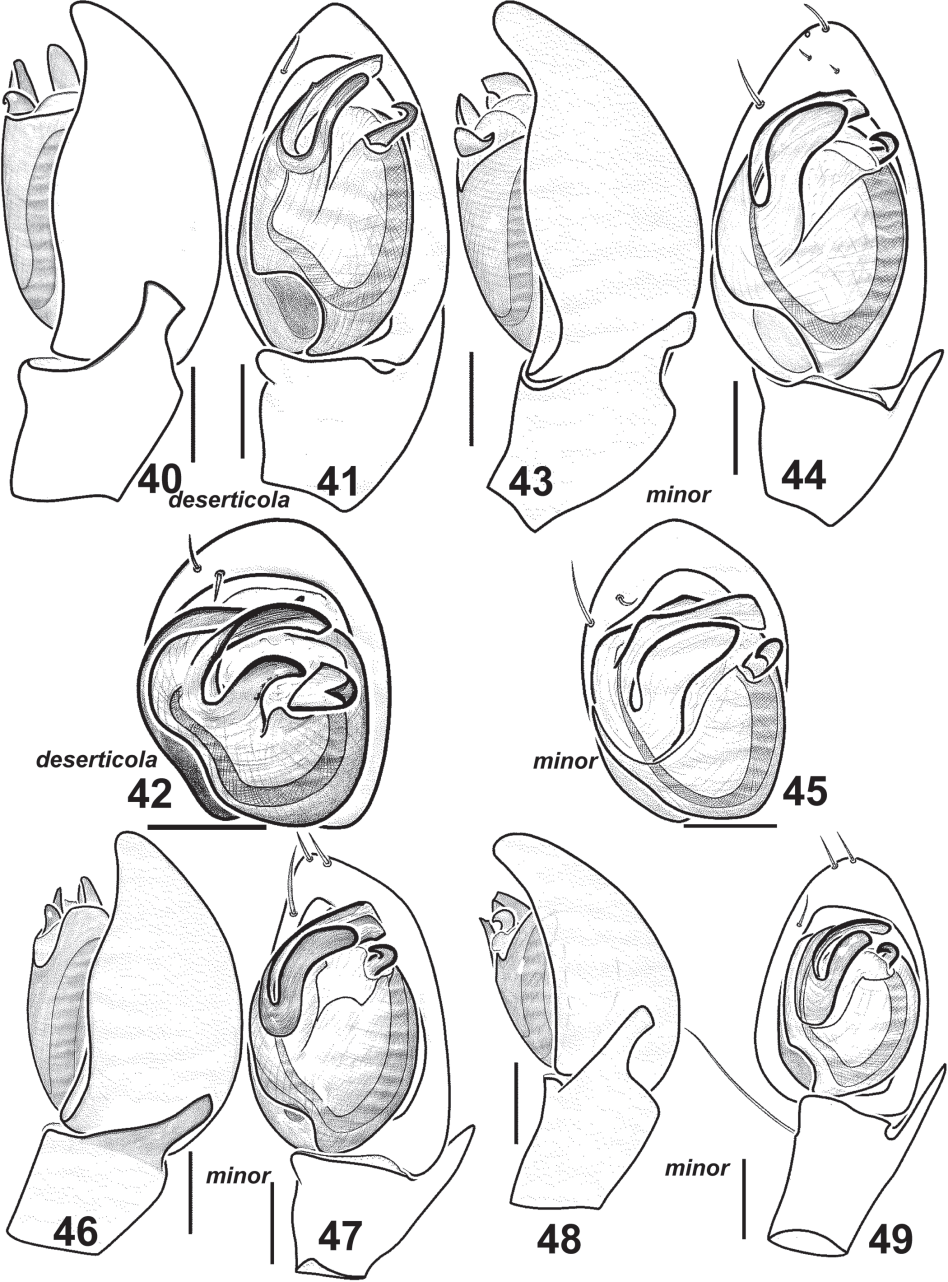
Males of *Haplodrassus deserticola* (**40–42** from Canary Islands) and *Haplodrassus minor* (4**3–45** from Poland, **46–47** from Nikolaev Area, **48–49** from Orenburg Area): **40, 43, 46, 48** palp, retrolateral view **41, 44, 47, 49** palp, ventral view **42, 45** bulbus, apical view.

**Figures 50–53.**
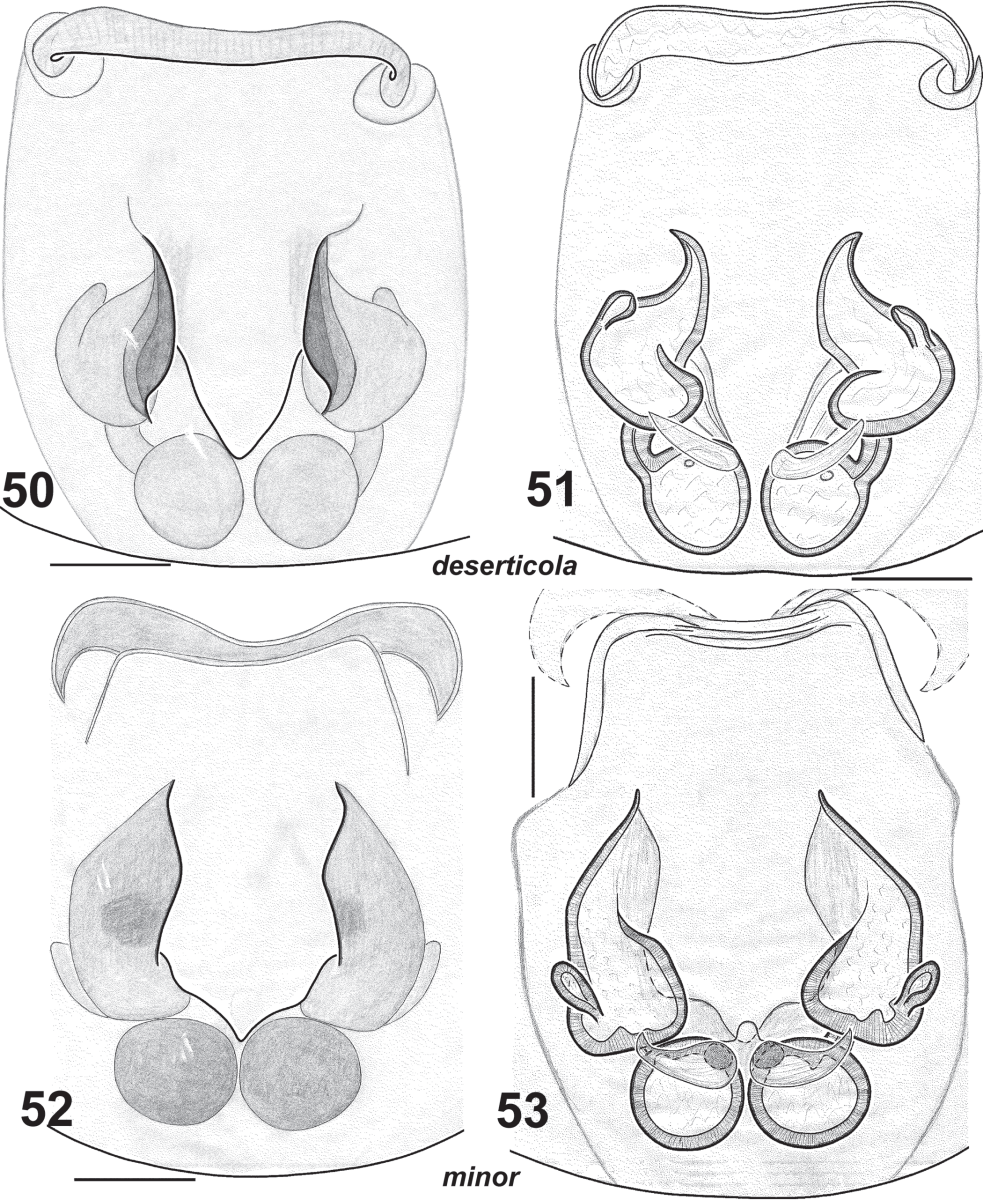
Females of *Haplodrassus deserticola* (**50–51** from the Canary Islands) and *Haplodrassus minor* (**52–53** from Nikolaev Area): **50, 52** epigyne, ventral view **51, 53** epigyne, dorsal view.

This species is most similar to *Haplodrassus deserticola* from the Canary Islands (Figs 40–42, 50–51). *Haplodrassus deserticola* differs from *Haplodrassus minor* by having a dorsal abdominal pattern (Fig. 66). *Haplodrassus minor* and *Haplodrassus deserticola* also differ by the shape of the embolus (thick in *Haplodrassus minor*, andthin in *Haplodrassus deserticola*), terminal apophysis (thick in *Haplodrassus minor*, and thin in *Haplodrassus deserticola*), median apophysis (short in *Haplodrassus minor*, and long in *Haplodrassus deserticola*), epigyne and spermathecae.

**Figures 54–63. F10:**
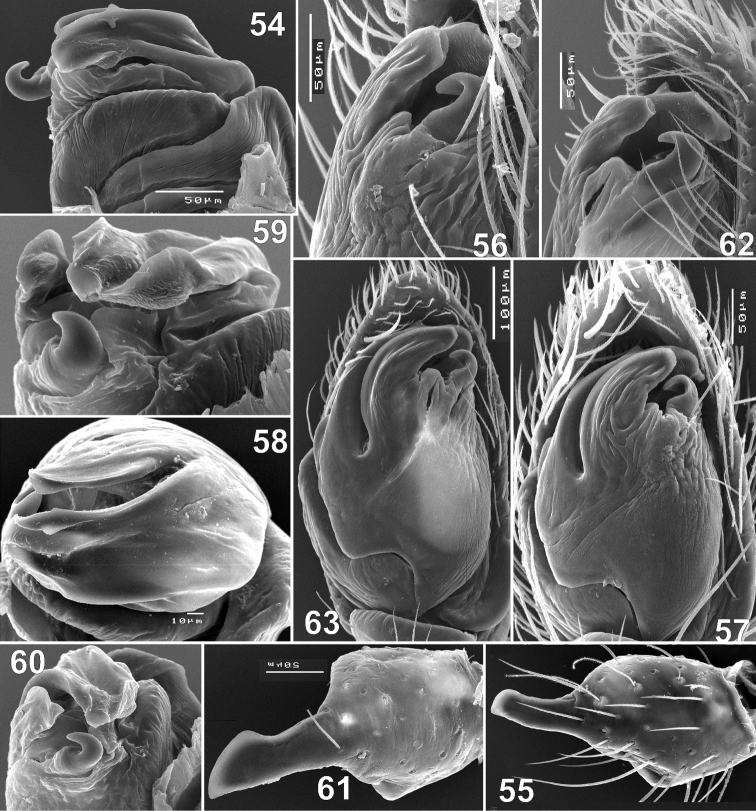
Males of *Haplodrassus minor* (**54–57** from Crimea, **58–63** from Orenburg Area): **54** apical part of bulbus, dorsal view **55, 61** tibia of palp, retrolateral view **56, 62** bulbus, retrolateral view **57, 63** bulbus, ventral view **58** bulbus, apical view **59** bulbus, retrolateral-dorsal view **60** apical part of bulbus, retrolateral view.

**Figures 64–67.**
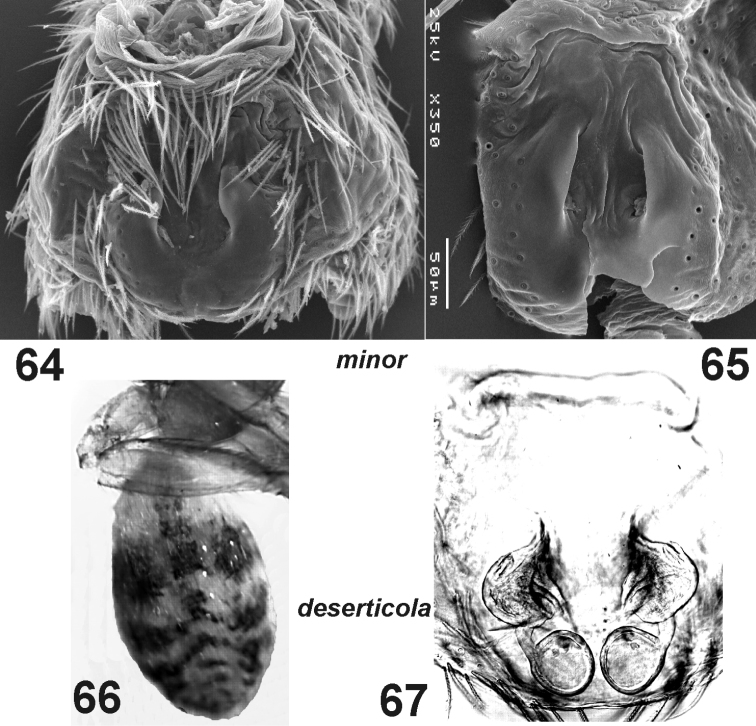
*Haplodrassus minor* (**64** from Crimea, **65** from Orenburg Area) and *Haplodrassus deserticola* (**66–67** from the Canary Islands): **64–65** epigyne, ventral view **66** male abdomen, dorsal view **67** epigyne, dorsal view.

#### Variations.

*Haplodrassus minor* ishighly variable in body size and also in the shape of the bulbal apophyses, RTA (slightly indented or not indented) and the epigyne (Figs 43–65). It is interesting to note that the width of the terminal apophysis decreases with increasing longitude (i.e. from west to east) (cf. Figs 44, 47, 49, 57, 63).

#### Distribution.

The species has a European range and is known from Portugal to Ural, north to Wales and south to Crete (Tuneva, Esyunin 2003; Helsdingen 2010; Platnick 2012).

#### Habitats.

Steppes, meadows, forest strips (=shelterbelts) within steppes.

#### Phenology.

In Crimea ♂♀ – V-VI, ♂♂ – IV, ♀♀ – VII, the peak of activity in adults occurs in May. In Britain ♂♀ – IV-VI, with the peak in June (Harvey et al. 2002), a month later than in Crimea.

### Haplodrassus pseudosignifer

Marusik, Hippa & Koponen, 1996

http://species-id.net/wiki/Haplodrassus_pseudosignifer

Figs 68–70 75–76

Haplodrassus pseudosignifer Marusik et al., 1996: 26, f. 63–65, 69 (♂♀).

#### Type material.

RUSSIA. 2 ♂♂ paratypes (ZMT), SW Altai, 7 km W Katanda, Katun River valley, forest steppe, pitfall traps, 22.06.–26.07.1983, H. Hippa.

#### Material.

UKRAINE, CRIMEA: Feodosiya Distr.:19 ♂♂, 6 ♀♀ (TNU), Karadag Nature Reserve, 15.10.2006–05.2011, M.M. Kovblyuk, O.V. Kukushkin, A.A. Nadolny. Simferopol Distr.: 2 ♂♂, 2 ♀♀ (TNU), Bayrakly Mt. (519 m), ~ 400 m, 14.05.–23.06.2000, M.M. Kovblyuk; 1 ♂ (TNU), Chatyr-Dag Mt., east slope, 10–28.06.2000, M.M. Kovblyuk; 1 ♀ (TNU), near Skvortsovo Vill., 9–30.06.2002, M.M. Kovblyuk. Sudak Distr.: 28 ♂♂, 7 ♀♀ (TNU), 10 km west from Sudak Town, Mezhdurechie Vill., 3.05.–3.06.2010, M.K. Yusufova. Yalta Distr.: 1 ♂ (TNU), Ay-Petri Yaila Mt., west part, 12–13.06.1999, O.V. Kukushkin; 15 ♂♂, 1 ♀ (TNU), Nikitskaya Yaila Mt. (=Skrinita), ~ 1200 m, 2.06.–24.07.2001, M.M. Kovblyuk.

#### Additional material.

UKRAINE. Nikolaev Area: 1 ♀ (TNU), Pervomaysky Distr., Kuripchane Vil., 26.05–8.06.2006, N.Yu. Polchaninova.

#### Note.

Identification of this species was based on comparison of our specimens with the male paratypes from ZMT. Specimens from Crimea and Altai differ only slightly in the shape of the tooth on embolus. In our opinion the specimens from Crimea, Nikolaev Area and Altai are conspecific.

#### Diagnosis.

*Haplodrassus pseudosignifer* is very similar to *Haplodrassus signifer*. The two species have no distinct differences in coloration, size or leg spination, but *Haplodrassus pseudosignifer* can be differentiated from *Haplodrassus signifer* by having an almost straight and shorter terminal apophysis and thinner embolus, and by the shape of the lateral pockets and fovea of the epigyne.

#### Description.

Males (n = 5) and females (n = 5). Measurements (♂ / ♀): total length 5.7–8.0 (6.79) / 6.6–10.5 (7.8); carapace 2.8–3.5 (3.1) / 2.6–3.6 (3.3) long, 2.2–2.6 (2.4) / 2.1–3.0 (2.6) wide; abdomen 3.1–4.8 (3.8) / 3.4–6.7 (4.7) long, 1.7–2.4 (2.0) / 2.1–4.2 (2.8) wide.

Length of leg segments:

**Table:** 

**Leg**	**Femur**	**Patella**	**Tibia**	**Metatarsus**	**Tarsus**	**Total**
I♂	1.9–2.6 (2.3)	1.2–1.6 (1.4)	1.5–2.1 (1.8)	1.2–1.7 (1.5)	1.0–1.2 (1.1)	6.7–9.2 (8.4)
II♂	1.6–2.2 (2.0)	1.0–1.4 (1.2)	1.2–1.8 (1.5)	1.1–1.6 (1.3)	0.8–1.1 (1.0)	5.8–8.1 (7.0)
III♂	1.4–1.9 (1.7)	0.7–1.0 (0.9)	0.9–1.2 (1.0)	1.2–1.5 (1.4)	0.8–1.1 (0.9)	5.2–6.7 (5.9)
IV♂	2.0–2.6 (2.3)	1.0–1.4 (1.2)	1.5–2.02 (1.8)	1.7–2.4 (2.0)	1.0–1.3 (1.1)	7.2–9.8 (8.5)
I♀	1.8–2.4 (2.2)	1.0–1.5 (1.4)	1.4–1.9 (1.7)	1.1–1.5 (1.3)	0.8–1.1 (1.0)	6.2–8.4 (7.5)
II♀	1.6–2.2 (1.9)	1.0–1.4 (1.2)	1.1–1.6 (1.4)	1.0–1.4 (1.2)	0.8–1.0 (0.9)	5.6–7.5 (6.7)
III♀	1.4–1.9 (1.7)	0.8–1.0 (0.9)	0.8–1.2 (1.0)	1.1–1.5 (1.3)	0.7–1.0 (0.9)	4.9–6.6 (5.8)
IV♀	2.0–2.6 (2.4)	1.1–1.4 (1.3)	1.4–2.0 (1.8)	1.6–2.2 (2.0)	0.9–1.2 (1.1)	7.0–9.5 (8.5)

Length of palp segments (male / female): femur 1.0–1.3 (1.2) / 0.8–1.2 (1.1), patella 0.4–0.7 (0.5) / 0.5–0.6 (0.5), tibia 0.3–0.4 (0.38) / 0.4–0.6 (0.5), tarsus 1.0–1.2 (1.1) / 0.7–0.9 (0.8).

Chelicerae with 2–3 promarginal and 2 retromarginal teeth in males and females. Number of promarginal teeth varies from 2 (frequently) to 3 (rarely).

Coloration grey.

**Figures 68–74.**
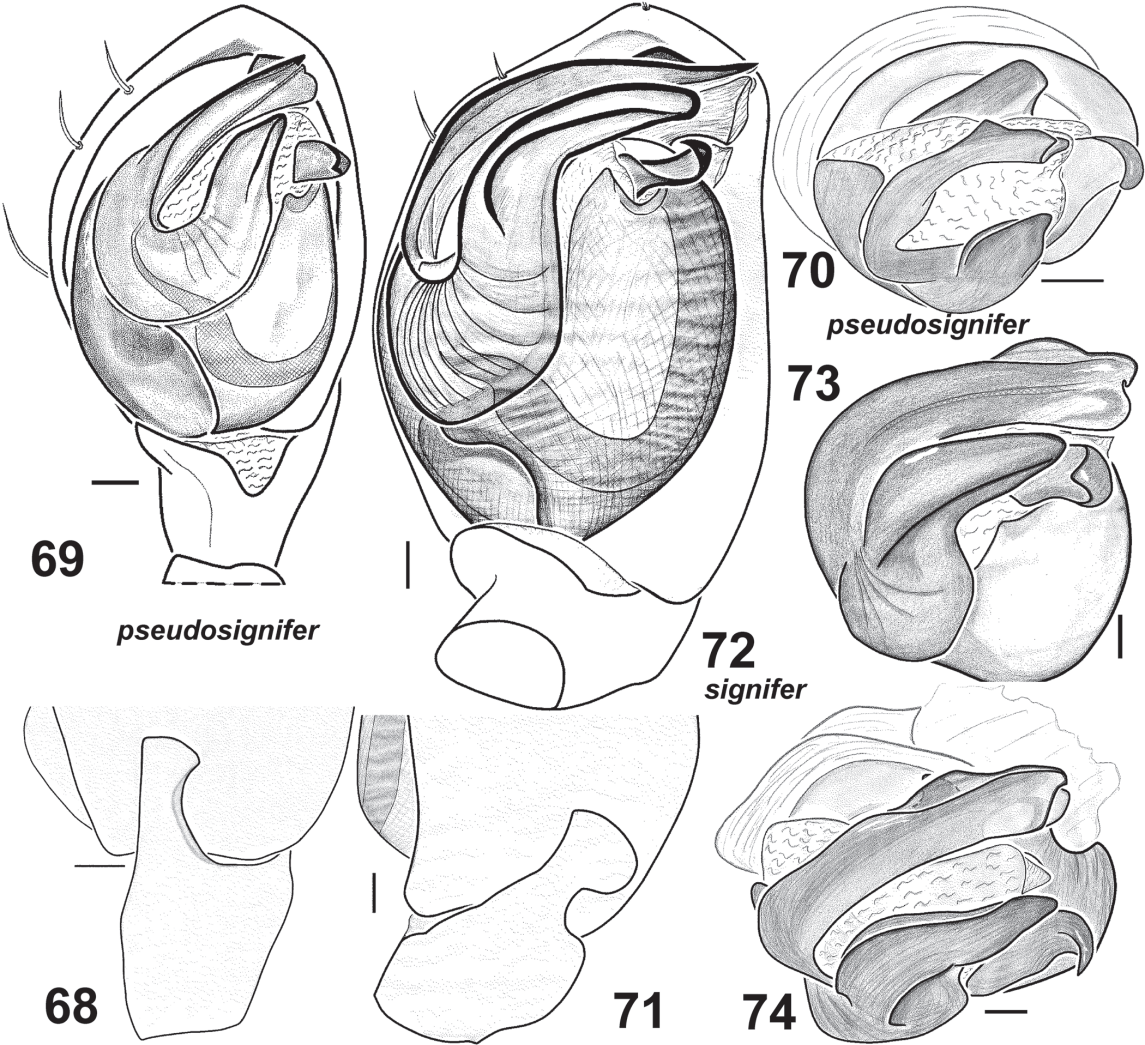
Males of *Haplodrassus pseudosignifer* (**68–70** from Crimea) and *Haplodrassus signifer* (**71–74** from Crimea): **68, 71** RTA, retrolateral view **69, 72** palp, ventral view **70, 73–74** bulbus, apical view.

Male palp as in Figs 68–70. Terminal apophysis short (length/width ratio ~ 2) and straight, ridge poorly developed; embolus almost straight and with a tooth.

**Figures 75–78.**
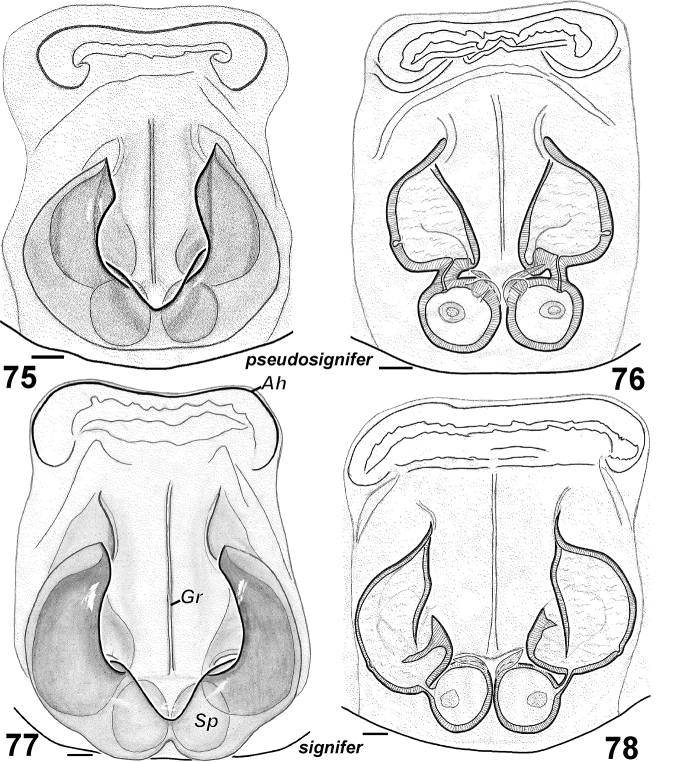
Females of *Haplodrassus pseudosignifer* (**75–76** from Crimea) and *Haplodrassus signifer* (**77–78** from Crimea): **75, 77** epigyne, ventral view **76, 78** epigyne, dorsal view. Abbreviations: ***Ah*** anterior hood; ***Gr**** *groove of epigyne; ***Sp***spermatheca.

Epigyne as in Figs 75–76. Fovea elongated, rectangular (longer than wide) with narrow longitudinal groove.

#### Distribution.

Steppe zone of Eurasia: from Crimea and Nikolaev Area of Ukraine east to Altai (Marusik et al. 1996; present data).

#### Comments.

*Haplodrassus pseudosignifer* is a new species record for Crimea and Ukraine. Crimea is the westernmost point of the known distribution range.

#### Habitats.

Forests with *Pistaca mutica* or *Quercus pubescens*, forest-steppes, forest stripes (=shelter belts), rocky steppes, grasslands.

#### Phenology.

♂♀ – V-VII, ♂♂ – IV, X. In Crimea the peak of activity in adults occurs in May.

### Haplodrassus signifer

(C.L. Koch, 1839)

http://species-id.net/wiki/Haplodrassus_signifer

Figs 71–74 77–78

Haplodrassus signifer : Tullgren 1946: 98, f. 30A, pl. 16, f. 197–200 (♂♀).
Haplodrassus signifer : Miller and Buchar 1977: 168, pl. II, f. 7–10 (♂♀).
Haplodrassus signifer : Thaler 1984: 189, f. 9d–f, i (♂).
Haplodrassus signifer : Grimm 1985: 146, f. 146–148, 170–171 (♂♀).
Haplodrassus signifer : Roberts 1985: 66, f. 23g (♂♀).
Haplodrassus signifer : Marusik et al. 1996: 26, f. 66–68, 70 (♂♀).
Haplodrassus signifer : Roberts 1998: 109, f. (♂♀).
Haplodrassus signifer : Levy 2004: 19, f. 46–52 (mf).
Haplodrassus signifer : Songet al. 2004: 139, f. 82A–I (♂♀).
Haplodrassus signifer : Almquist 2006: 411, f. 354a–i (♂♀). For a complete list of references see Platnick (2012).

#### Records from Crimea.

Thorell (1875) – sub *Drassus troglodytes* C.L. Koch; Spassky (1927) – sub *Drassodes*; Charitonov (1932) – sub *Drassodes*; Ovtsharenko (1982); Mikhailov (1997); Kovblyuk (2001, 2004, 2006); Kovblyuk and Kukushkin (2007); Kovblyuk et al. (2008).

#### Material.

UKRAINE, CRIMEA:Feodosiya Distr.:5 ♂♂, 2 ♀♀ (TNU), Karadag Nature Reserve, 27.04.2004.–23.05.2008, M.M. Kovblyuk, O.V. Kukushkin. Saky Distr.: 121 ♂♂, 27 ♀♀ (TNU), near Pribrezhnaya railway station, 16.04.–24.06.2000, M.M. Kovblyuk. Simferopol Distr.: 2 ♂♂ (EMZ), near Simferopol water reservoir, 2.05.1997, M.M. Kovblyuk & G.V. Reutov; 1 ♀ (EMZ), near Strogonovka Vill., 16.05.1997, M.M. Kovblyuk & S. Dyadyushkin; 46 ♂♂, 1 ♀ (EMZ), near Kirpichnoe Vill., 14.05.–12.06.1997, M.M. Kovblyuk; 1 ♀ (TNU), near Fersmanovo Vill., Kesslers’ forest, 350–400 m, 6–23.06.2000, M.M. Kovblyuk; 1 ♂ (TNU), Bayrakly Mt. (519 m), ~ 400 m, 26.05.–6.06.2000, M.M. Kovblyuk; 3 ♂♂, 2 ♀♀ (TNU), near Lozovoe Vill., ~ 250 m, 1.05.–6.06.2000, M.M. Kovblyuk; 8 ♂♂ (TNU), near Simferopol water reservoir, 1.05.–23.06.2000, M.M. Kovblyuk; 2 ♂♂, 1 ♀ (TNU), Simferopol, Bitak Mt., 3.05.–6.06.2000, M.M. Kovblyuk; 4 ♂♂ (TNU), Chatyr-Dag Mt., 20–17.07.2000, M.M. Kovblyuk; 267 ♂♂, 51 ♀♀ (TNU), near Skvortsovo Vill., 12.03.–30.06.2002, M.M. Kovblyuk; 1 ♀ (TNU), Lozovoe Vill., 18.05.2006, M.M. Kovblyuk. Sudak Distr.: 9 ♂♂ (TNU), 10 km W from Sudak Town, Mezhdurechie Vill., 6.05.–22.05.2010, M.K. Yusufova. Yalta Distr.: 5 ♂♂, 3 ♀♀ (TNU), 1 km N Nikita Vill., 22.04.–30.05.2000, M.M. Kovblyuk; 1 ♂, 2 ♀♀ (YMC), Nikitskaya Yaila Mt. range, (=Skrinita), ~ 1200 m, 2.06.–3.07.2001, M.M. Kovblyuk; 2 ♂♂ (TNU), Yalta Mountain-Forest Natural Reserve, near Upper Nikita Lake, 28.04–12.05.2002, A.A. Khaustov; 2 ♂♂, 2 ♀♀ (TNU), near Nikita Vill., 13–26.05.2002, A.A. Khaustov.

#### Additional material.

SLOVAKIA. 2 ♂♂, 2 ♀♀ (TNU), Nova’ky, 11.05.1990, S. Pekar. UKRAINE. Chernovtsy Area:1 ♀ (TNU), Tsetsino Town, 8–19.05.2009, V.V. Garashchuk, T.O. Auzyak. Donetsk Area: 1 ♀ (TNU), Slavyansky Distr., Svyatogorsk Town, N49°02', E37°39', 07.2004, N.Yu. Polchaninova. Kherson Area: 2 ♂♂, 3 ♀♀ (TNU), Henichesk Distr., Arabatskaya strelka, 4 km S Henichesk Town, 23–30.05.2011, N.A. Stasyuk. RUSSIA. Leningrad Area: 3 ♂♂, 2 ♀♀ (TNU), Nizhnesvirskyi Reserve, 21.07.1994, T.I. Oliger. Kursk Area: 2 ♂♂, 1 ♀ (TNU), Medvensky Distr., “Kazatskaya Steppe” Department of the Tsentral’no-Chernozemny Nature Reserve, 51°30'N, 36°17'E, 20.05.–30.06.2004, N.Yu. Polchaninova. Rostov Area: 8 ♂♂, 1 ♀ (TNU from CP), Orlovsky Distr., Rostovsky Narure Reserve, Starikovskyi region, 15.06.2003 & 7.05.2004, Z.G. Prishutova. ABKHAZIA. Gagra Distr.: 3 ♀♀ (TNU), Gagra Distr., Gagra Range, Mamdzyshkha Mt. (1866 m), from border of forest (43°18'N, 40°19'E, 1705 m) to the peak, wood (*Abies*, *Fagus*, *Acer*) and alpine meadows, 7–15.07.2009, M.M. Kovblyuk.

#### Diagnosis.

The species can be easily recognized by the shape of the terminal apophysis, which has a peculiar long ridge, and also by the shape of the epigyne. *Haplodrassus signifer* is very similar to *Haplodrassus pseudosignifer* (see the diagnosisfor *Haplodrassus pseudosignifer*).

#### Distribution.

Circum-Holarctic polyzonal range (Marusik et al. 2000; Platnick 2012).

#### Habitats.

Steppes, meadows, shrubby communities, forests.

#### Phenology.

♂♀ – IV-VII, ♂♂ – III. In Crimea the peak activity in adults’ occurs in May. In Central Europe ♂♀ – IV-XII (Nentwig et al. 2011).

#### Comments.

*Haplodrassus signifer* is the largest and most abundant *Haplodrassus* species in Crimea.

### Haplodrassus umbratilis

(L. Koch, 1866)

http://species-id.net/wiki/Haplodrassus_umbratilis

igs 79–83

Haplodrassus umbratilis : Tullgren 1946: 101, f. 30B, pl. 16, f. 204–208 (♂♀).
Haplodrassus umbratilis : Miller and Buchar 1977: 168, pl. III, f. 2–4 (♂♀).
Haplodrassus umbratilis : Grimm 1985: 156, f. 150, 158–159 (♂♀).
Haplodrassus umbratilis : Roberts 1985: 66, f. 25a (♂♀).
Haplodrassus umbratilis : Roberts 1998: 112, f. (♂♀).
Haplodrassus umbratilis : Almquist 2006: 413, f. 357a-f (♂♀). For a complete list of references see Platnick (2012).

#### Records from Crimea.

Kovblyuk (2006).

#### Material.

Ukraine. CRIMEA. Feodosiya Distr.: 2 ♂♂, 1 ♀ (TNU), Karadag Nature Reserve, thalweg Karadag beams, 44°55'11.4"N, 35°12'25.5"E, 43 m, 9.05.–6.06.2008, M.M. Kovblyuk. Simferopol Distr.: 1 ♀ (TNU), 2 km N Pionerskoe Vill., 10.06.1998, M.M. Kovblyuk; 4 ♂♂ (TNU), 1,5 km NE Fersmanovo Vill., Kesslers’ forest, 14.05.–6.06.2000, M.M. Kovblyuk; 64 ♂♂, 15 ♀♀ (TNU), Chatyr-Dag Mt., 23.04.–2.09.2000, M.M. Kovblyuk. Sudak Distr.: 1 ♂ (TNU), 10 km W Sudak Town, Mezhdurechie Vill., 6–8.05.2010, M.K. Yusufova.Yalta Distr.: 68 ♂♂, 27 ♀♀ (TNU), Nikitskaya Yaila Mt. (=Skrinita), ~ 1200 m, 4.05.–10.11.2001, M.M. Kovblyuk.

#### Additional material.

UKRAINE. Donetsk Area: 5 ♀♀ (TNU), Slavyansky Distr., Svyatogorsk Town, N49°02', E37°39', 8–30.06.2004, N.Yu. Polchaninova. Kherson Area:6 ♂♂, 4 ♀♀ (TNU), Golopristynskiy Distr., Chernomorskiy Reserve, Rybalchie Vill., 04–08.1989, Zelinskaya.

#### Comparative material.

*Haplodrassus soerenseni* (Strand, 1900): UKRAINE. Sumy Area: 1 ♀ (TNU), Vakolovshchina Vill., 1.06.1990, V.A. Gnelitsa.

#### Diagnosis.

*Haplodrassus umbratilis* can be easily differentiated by from all other *Haplodrassus* species found in Crimea by its terminal apophysis, which has a broad process (*Bp*). From the similar *Haplodrassus soerenseni* males differ in the shape of the terminal apophysis and embolus, and females by having longer lateral epigynal pockets.

**Figures 79–83.**
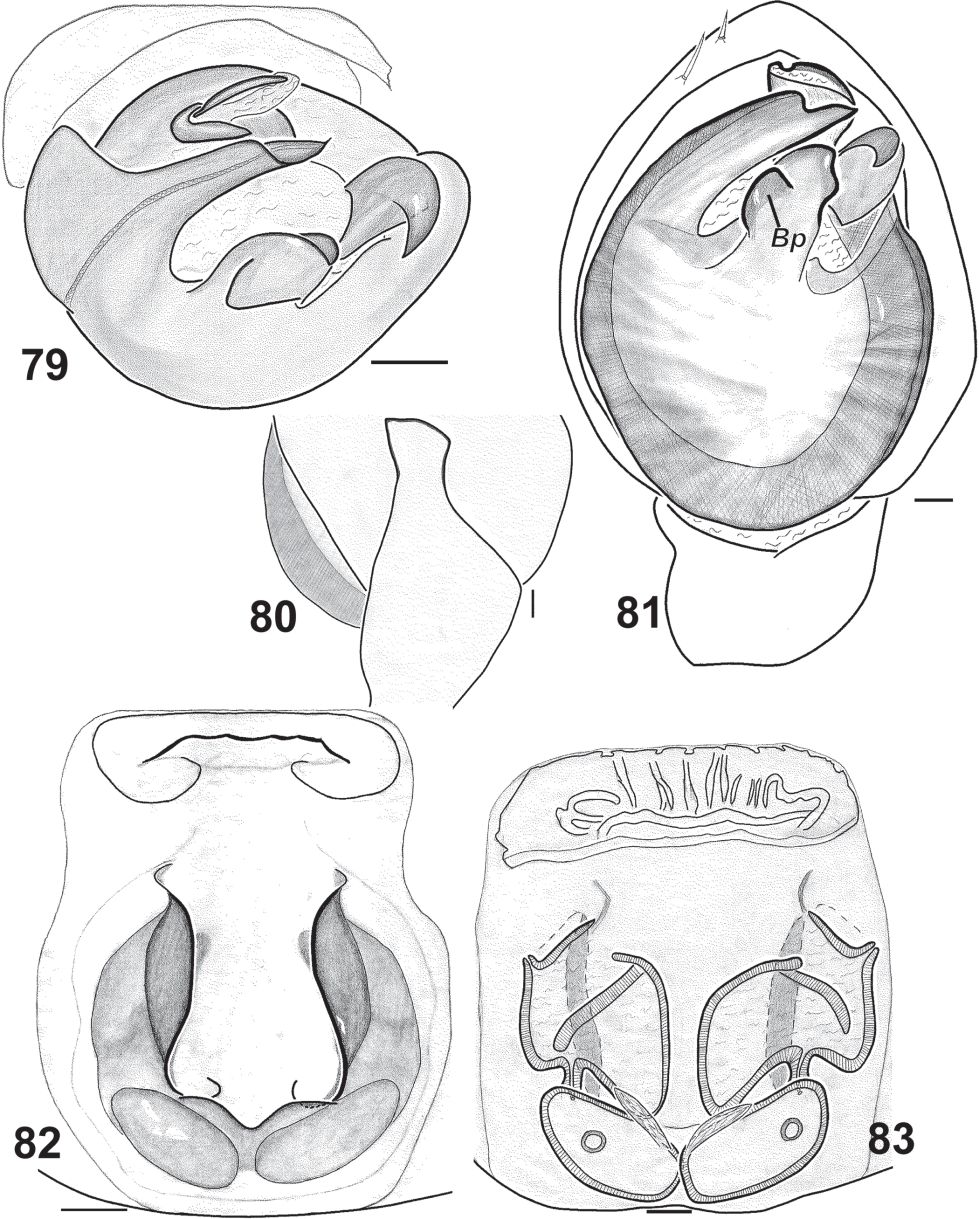
*Haplodrassus umbratilis* from Crimea: **79** bulbus, apical view **80** RTA, retrolateral view **81** palp, ventral view **82** epigyne, ventral view **83** epigyne, dorsal view. Abbreviations: ***Bp*** broad process of terminal apophysis.

#### Distribution.

West and Central Palaearctic: all Europe, Turkey, Caucasus, mountains of Central Asia and South Siberia (Mikhailov 1997; Helsdingen 2010; Platnick 2012).

#### Habitats.

Dry forests, forest edges, meadows and steppes.

#### Phenology.

♂♀ – V-VII, ♂♂ – IV, ♀♀ – VIII, X, the peak of activity in adults occurs in June. In Central Europe ♂♀ –VI-VIII (Nentwig et al. 2011). In Britain, the peak is in May (Harvey et al. 2002), a month earlier than in Crimea.

## Discussion

### Species diversity

The number of *Haplodrassus* species found in Crimea is rather high. Eight species, as in Crimea are known in the larger area of Bulgaria and Hungary (Helsdingen 2010). Several large countries such as Austria, Germany, Poland and Romania have 9 species of *Haplodrassus*. 10 species are known to occur in the Czech Republic, Switzerland and France. The highest species diversity in Europe, and probably in the Holarctic, is in Italy, with 13 species known from the mainland (Helsdingen 2010). Although Crimea was intensively investigated during only a short period we do not expect additional *Haplodrassus* species on the peninsula. The neighboring mainland Ukraine and Caucasus have no species that are absent in Crimea. There are several species that occurs in Romania or Bulgaria that are absent in Crimea: *Haplodrassus cognatus*, *Haplodrassus moderatus* (Kulczyński, 1897), and *Haplodrassus silvestris* (Blackwall, 1833). In terms of species diversity per unit area of a country or region, Crimea rates as the most diverse place in Europe and in the Mediterranean (8 species in ~ 26 000 km^2^). Only Israel has a similar number of species per unit area (8 species in ~27 000 km^2^).

## Phenology

Many specimens were collected using pitfall traps, which were regularly checked once in two week during one or two years. Thus, it was possible to analyze the seasonal dynamics of adult activity. All Crimean *Haplodrassus* species have one peak of activity of adults during the year. The maximum number of individuals and peak of activity for the adults of *Haplodrassus kulczynskii* occurred in April; for *Haplodrassus bohemicus*, *Haplodrassus minor*, *Haplodrassus pseudosignifer* and *Haplodrassus signifer* in May; for *Haplodrassus dalmatensis* and *Haplodrassus umbratilis* in June; for *Haplodrassus isaevi* in December. Probably all of the species studied have only one generation per year.

## Supplementary Material

XML Treatment for Haplodrassus

XML Treatment for Haplodrassus bohemicus

XML Treatment for Haplodrassus cognatus

XML Treatment for Haplodrassus dalmatensis

XML Treatment for Haplodrassus invalidus

XML Treatment for Haplodrassus isaevi

XML Treatment for Haplodrassus kulczynskii

XML Treatment for Haplodrassus minor

XML Treatment for Haplodrassus pseudosignifer

XML Treatment for Haplodrassus signifer

XML Treatment for Haplodrassus umbratilis
